# Age, growth, and intrinsic sensitivity of Endangered Spinetail Devil Ray (*Mobula mobular*) and Bentfin Devil Ray (*M. thurstoni*) in the Indian Ocean

**DOI:** 10.1007/s00227-024-04564-6

**Published:** 2024-12-30

**Authors:** Ellen Barrowclift, Andrew J. Temple, Sebastián A. Pardo, Alexander M. A. Khan, Shoaib Abdul Razzaque, Nina Wambiji, Mochamad Rudyansyah Ismail, Lantun Paradhita Dewanti, Per Berggren

**Affiliations:** 1 School of Natural and Environmental Sciences, Newcastle University, Newcastle-upon-Tyne, UK; 2 King Abdullah University of Science and Technology, Thuwal, Saudi Arabia; 3 Pacific Biological Station, Fisheries and Oceans Canada, Nanaimo, BC Canada; 4 Faculty of Fisheries and Marine Sciences, Universitas Padjadjaran, Bandung, Indonesia; 5 WWF Pakistan, Karachi, Pakistan; 6 Kenya Marine and Fisheries Research Institute, Mombasa, Kenya

**Keywords:** Life history, Demography, Small-scale fisheries, Elasmobranch, Batoids, Bayesian

## Abstract

Devil rays (*Mobula* spp.) are caught in fisheries across the Indian Ocean, with reports of significant recent declines in catch and sightings. Globally, the few populations studied have extremely low population growth rates due to low fecundity and long reproductive cycles, making them highly vulnerable to overfishing. To allow for assessment of the current sustainability of devil ray catch in the Indian Ocean, we provide estimates of age using the caudal vertebrae; somatic growth using a Bayesian, multi-model approach; maximum intrinsic rate of population increase (*r*_*max*_); and fishing mortality for Endangered Spinetail Devil Ray (*Mobula mobular*) and Bentfin Devil Ray (*M. thurstoni*) sampled from small-scale fisheries catch in Indonesia, Kenya, and Pakistan. The oldest individuals of Spinetail Devil Ray (*n* = 79) and Bentfin Devil Ray (*n* = 59) were 17.5 and six years, respectively. Both species had relatively low growth coefficients (*k* = 0.05 and *g* = 0.19 year^−1^, respectively), with the von Bertalanffy and logistic models providing the best fitting growth models, and low *r*_*max*_ (0.109 and 0.107 year^−1^, respectively) indicating that they are highly sensitive to overexploitation. Fishing mortality *F* estimates (0.16 and 0.18 year^−1^, respectively) were higher than *r*_*max*_ and exploitation ratio *E* (0.77 and 0.80, respectively) were higher than an optimum value of 0.5 for biological sustainability for both species, suggesting that the fisheries catches of the species are unsustainable. We demonstrate an approach to assess data-poor species and apply this to two Indian Ocean devil ray species. The results highlight the urgent need for better management actions to reduce the catch of all devil rays to prevent species extinction and aid in population recovery.

**Supplementary Information:**

The online version contains supplementary material available at 10.1007/s00227-024-04564-6.

## Introduction

Sharks and rays (class Chondrichthyes, subclass Elasmobranchii) generally exhibit slower growth, later sexual maturity, and lower fecundity than their teleost counterparts (Compagno 1990; Cortés 2000; Gravel et al. 2024). These life history traits result in lower population growth rates that restrict recovery potential (Cortés 2002; Dulvy and Forrest 2010) and make many species intrinsically sensitive to fisheries exploitation (García et al. 2008; Quetglas et al. 2016). Approximately 37% of chondrichthyans are threatened with extinction due to overfishing (Dulvy et al. 2021). Variations in life history among and within species, coupled with different exploitation rates, results in differences in fisheries resilience and localised extinction risk (Lombardi-Carlson et al. 2003; Jacobsen and Bennett 2010; Trinnie et al. 2014). Data on species and population-specific life history traits are therefore critical in predicting extinction risk and rebound potential, demographic modelling, fisheries stock assessments, and achieving sustainable fisheries management and global conservation goals (Frisk et al. 2001; Cortés 2002; Barnett et al. 2019).

Devil rays (*Mobula* spp., Family Mobulidae) are one of the most threatened chondrichthyan families (Dulvy et al. 2021). All devil ray species are listed on CITES (Convention on International Trade in Endangered Species of wild fauna and flora) Appendix II and CMS (Convention on the conservation of Migratory Species of wild animals) Appendices I and II, which regulate international trade and coordinate inter-governmental conservation efforts, respectively. Devil rays face high fisheries exploitation as target species and bycatch in both industrial and small-scale fisheries (Croll et al. 2016), exacerbated by an international market for their gill plates, which are used for food and traditional medicine in East Asia (Lawson et al. 2017; O’Malley et al. 2017). The Food and Agriculture Organisation of the United Nations (FAO) statistics indicate an annual global catch of over 4000 tonnes, a likely underestimate (Clarke et al. 2006; Pauly and Zeller 2016; FAO 2023). Devil rays have amongst the lowest maximum intrinsic rate of population increase (*r*_max_) and therefore highest intrinsic sensitivity to overfishing (Dulvy et al. 2014; Pardo et al. 2016a; Rambahiniarison et al. 2018). This sensitivity is in part due to their extremely low fecundity (Pardo et al. 2018), with species in this genus known to produce only a single pup per litter (rarely twins) every 1–7 years, following a 12-month gestation period (Last et al. 2016; Stevens et al. 2018). These life history traits have been observed in only a handful of studies and locations for these circumglobal, tropical and warm-temperate species (Notarbartolo di Sciara 1988; Villavicencio-Garayzar 1991; Marshall and Bennett 2010; Doumbouya 2011; Kashiwagi 2014; Stevens 2016; Ehemann et al. 2017; Broadhurst et al. 2018, 2019).

Despite there being several studies on devil ray life histories, there has only been a single aging study to date (Cuevas-Zimbrón et al. 2013). Given the importance of demographic data in assessing fisheries sustainability (Musick and Bonfil 2005) and considering the broad distributions of some devil ray species (Couturier et al. 2012), further understanding of species and population-specific life history parameters are needed for effective evidence-based management (Barnett et al. 2019). Available evidence suggests that devil rays that inhabit coastal and continental shelf waters may exhibit genetic population structuring, including Shorthorned Pygmy Devil Ray (*Mobula kuhlii*) and Reef Manta Ray (*Mobula alfredi*) between the eastern and western Indian Ocean as well as Spinetail Devil Ray (*Mobula mobular*) and Reef Manta Ray between the Indian Ocean and the Pacific (Venables et al. 2020; Hosegood et al. 2020; Lassauce et al. 2022; Humble et al. 2023). However, highly migratory and more offshore species including Bentfin Devil Ray (*Mobula thurstoni*) and Oceanice Manta Ray (*Mobula birostris*) show no evidence of population structuring, potentially due to more opportunity for gene flow (Hosegood et al. 2020; Humble et al. 2023). The extent of genetic population structuring and connectivity will have implications for the status of devil ray species and populations and can inform the most effective conservation actions.

Countries in the Indian Ocean region are among those reporting the highest devil ray catches (Couturier et al. 2012; Ward-Paige et al. 2013; Croll et al. 2016; Lawson et al. 2017). Six of the seven devil ray species in the Indian Ocean are listed as Endangered on the IUCN Red List; the Vulnerable Reef Manta Ray being the only exception (IUCN 2024). Devil rays are commonly caught in small-scale fisheries, primarily in gillnets, that provide important sources of protein and income for coastal communities, particularly in low-income countries (Temple et al. 2019, 2024; Flounders 2020). Devil rays are often accidental catch and in some locations such as Pakistan, they will be released or used for fish meal (Razzaque pers. obs.). However, in other locations, even where devil rays are not target catch in small-scale coastal and offshore gillnet fisheries, they will often be retained for their meat and gill plates due to their high value (White et al. 2006; Moazzam 2018; Martin 2020). Devil rays are also caught in industrial tuna fisheries, mainly utilising purse-seine but also in longline and drift gillnet fishing gears (Shahid et al. 2018; Flounders 2020). There is evidence of significant declines in sightings and fisheries catch (over 90%) in some locations across the Indian Ocean (Lewis et al. 2015; Rohner et al. 2017; Moazzam 2018; Fernando and Stewart 2021; Carpenter et al. 2023). Consequently, devil rays are amongst species of concern within the Indian Ocean Tuna Commission (IOTC) Area of Competence, which led the IOTC to adopt a resolution (19/03) for their conservation, including recommending the collection of species-specific data for fisheries catches (IOTC 2019). The IOTC resolution prohibits the retention of devil rays and encourages live release and research on post-capture mortality, although this does not apply to subsistence fisheries where rays are consumed locally by the fishers. There are also some national protections in place, for example, in Pakistan and for two manta ray species (Oceanic and Reef Manta Rays) in Indonesia, but many fisheries remain unregulated. Defining life history parameters can inform devil ray species assessments for sustainable management within the Indian Ocean region.

The aim of this study is to improve the knowledge of devil ray life histories by producing disc width-weight relationships, estimating age, growth, and maximum intrinsic rate of population increase (*r*_max_) for Spinetail Devil Ray and Bentfin Devil Ray caught in small-scale fisheries in Indonesia, Kenya, and Pakistan. We further use the disc width-at-age dataset to estimate total mortality, fishing mortality, and the exploitation ratio for the two species.

## Materials and methods

### Sample collection

**Fig. 1:**
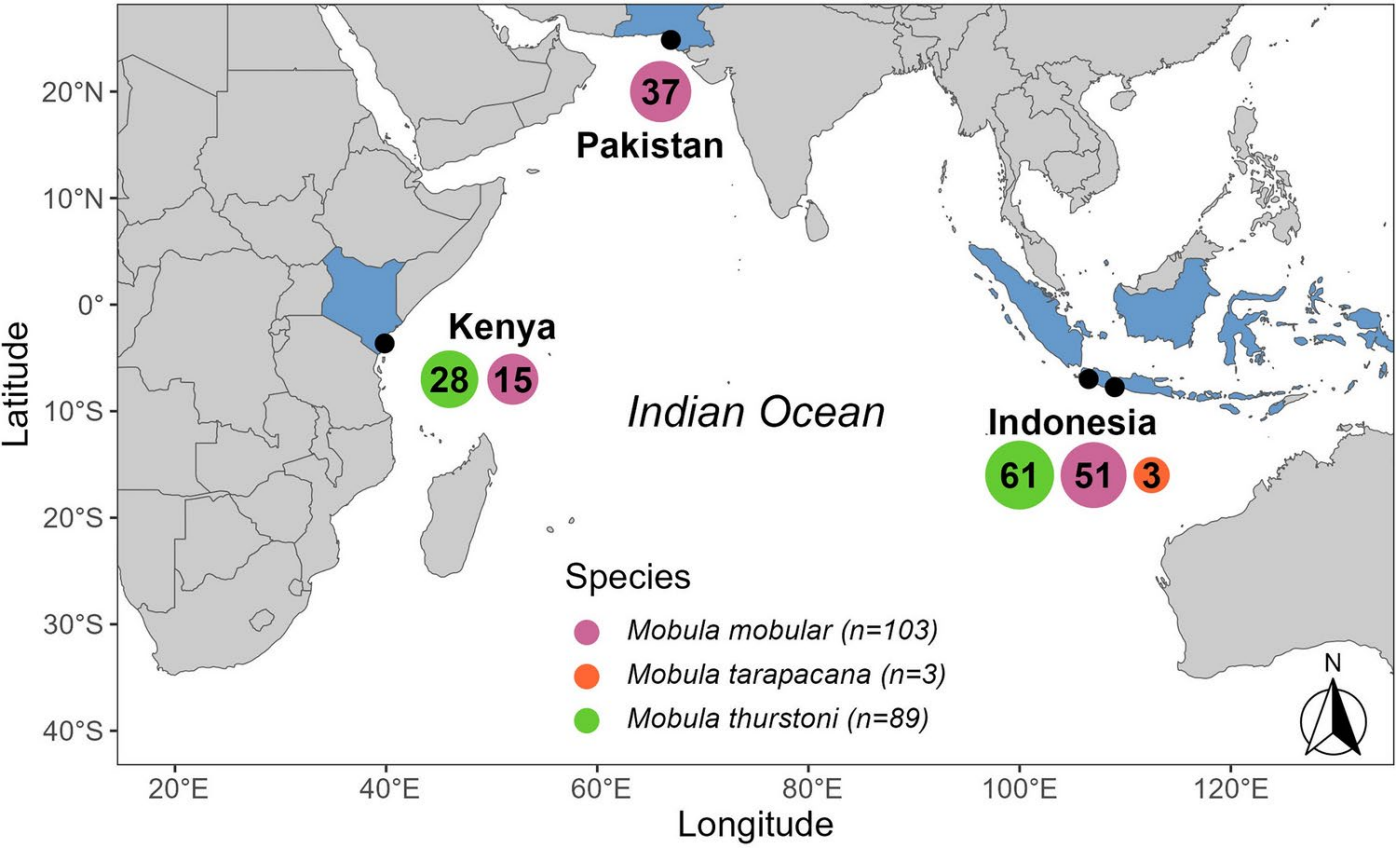
Sampling locations for *Mobula mobular*, *M. thurstoni*, and *M. tarapacana* across the Indian Ocean (*n* = 195)

Spinetail Devil Ray (hereafter, *M. mobular)* (*n* = 103) and Bentfin Devil Ray (hereafter, *M. thurstoni*) (*n* = 89) were opportunistically sampled from small-scale fisheries landing sites in Cilacap Fishing Port, Central Java (*n* = 15) and Palabuhanratu, West Java, Indonesia (*n* = 100) between September 2020 and December 2022; Kilifi Central, Kilifi, Kenya (*n* = 43) between February and March 2021; and Karachi Fish Harbour, Sindh, Pakistan (*n* = 37) between June 2021 and October 2022 with the consent of fishers and/or traders (Fig. 1). A further 3 individuals of Sicklefin devil ray (*M. tarapacana*) were sampled in Indonesia.

The sex, disc width (DW) in cm, and weight in kg of every individual were recorded. Where possible, vertebrae samples (*n* = 141) were taken from the caudal portion of the vertebral column for aging and were stored at − 20°C (Fig. S1a) (Cuevas-Zimbrón et al. 2013). Male maturity (immature or mature) was recorded based on the calcification of claspers, whereby only male specimens with fully calcified claspers were considered mature (Walker 2005). Where fishers and traders consented (*M. mobular*, *n* = 27; *M. thurstoni*, *n* = 6), female reproductive tracts were dissected to determine maturity, with females considered mature by the presence (mature) or absence (immature) of well-developed, yolky ova in the ovaries (Walker 2005). Individuals were caught in bottom-set and drift gillnets (*n* = 184) across the three countries, with additional catch in longline (*n* = 2), handline (*n* = 2), and purse seine (*n* = 4) in Indonesia.

## Disc width-weight relationship

Species- and location-specific DW frequency distributions were fit using 5 cm size bins for *M. mobular* and *M. thurstoni*. Bayesian linear models were fit to natural log (ln) transformed DW and weight data for *M. mobular* (*n* = 101) and *M. thurstoni* (*n* = 76) (Froese et al. 2014). Due to small sample sizes, all models were fit for each species across locations and for combined sex. Informative priors were constrained for *a* and *b* constants based on estimates available on FishBase (Froese and Pauly 2022), which was approximately 0.005 (− 5 for log(*a*)) and 3 for *a* and *b,* respectively (Table 1). We also compared the effect on posteriors with parameter estimates using weaker priors with the same mean of the distributions but higher variance (Table 1). A weakly informative prior is used for the variance σ^2^ in all models (Table 1).

**Table 1:** Strong and weaker priors used for each parameter in Bayesian length–weight regression, growth models, and length-maturity regression models for *Mobula mobular* (*n* = 79) and *M. thurstoni* (*n* = 59)

Species	Model	Parameter	Strong priors	Weaker priors
*M. mobular* and *M. thurstoni*	DW-weight linear regression	*b*	*normal*(3, 0.5)	*normal*(3, 1)
		*log(a)*	*normal*(− 5, 1)	*normal*(− 5, 3)
		σ^2^	*halfCauchy*(0, 30,000)	*halfCauchy*(0, 30,000)
	Age-maturity logistic regression	*β*	*normal*(0,10)	*normal*(0,10)
		*a*	*normal*(logit(0.5) + beta * α_mat_, 10)	*normal*(0,10)
	DW-maturity logistic regression	*β*	*normal*(0,10)	*normal*(0,10)
		*a*	*normal*(logit(0.5) + beta * DW_mat_, 10)	*normal*(0,10)
*M. mobular*	Growth models (von Bertalanffy, Gompertz, Logistic, Lester)	*k*	*uniform*(0, 2)	*uniform*(0, 2)
		*DW*_*0*_	*normal*(900, 200)	*normal*(900, 300)
		*DW*_*∞*_	*normal*(3500*kappa, 100)	*normal*(3500*kappa, 400)
		*kappa*	*gamma*(1000, 990)	*gamma*(200, 198)
		σ^2^	*halfCauchy*(0, 30,000)	*halfCauchy*(0, 30,000)
	Lester (biphasic) growth model	*T*	*normal*(4, 1)	*normal*(4, 4)
		*h*	*normal*(500, 1000)	*normal*(500, 1000)
		*t*_*1*_	*normal*(0, 20)	*normal*(0, 10)
		σ^2^	*halfCauchy*(0, 30,000)	*halfCauchy*(0, 30,000)
*M. thurstoni*	Growth models (von Bertalanffy, Gompertz, Logistic, Lester)	*k*	*uniform*(0, 2)	*uniform*(0, 2)
		*DW*_*0*_	*normal*(700, 200)	*normal*(700, 300)
		*DW*_*∞*_	*normal*(1970*kappa, 100)	*normal*(1970*kappa, 400)
		*kappa*	*gamma*(1000, 980)	*gamma*(200, 196)
		σ^2^	*halfCauchy*(0, 30,000)	*halfCauchy*(0, 30,000)
	Lester (biphasic) growth model	*T*	*normal*(4, 1)	*normal*(4, 4)
		*h*	*normal*(500, 1000)	*normal*(500, 1000)
		*t*_*1*_	*normal*(0, 5)	*normal*(0, 10)
		σ^2^	*halfCauchy*(0, 30,000)	*halfCauchy*(0, 30,000)

## Age estimation using caudal vertebrae

In line with Cuevas-Zimbrón et al. (2013), we found no vertebral centra in the thoracic portion of the vertebral column and that vertebrae size increased in the caudal portion of the vertebral column below the origin of the dorsal fin. We therefore sampled caudal vertebrae for age estimation (Fig. S1a in the supplementary materials). Neural and haemal arches along with excess tissue were removed from vertebrae samples using a scalpel. Vertebrae were subsequently placed into 5% diluted bleach for a maximum of five minutes depending on vertebrae size to remove any remaining connective tissue and then rinsed with distilled water. Cleaned vertebrae centra samples were left to air dry overnight. Vertebrae centra were submerged in a mixture of EpoxiCure 2 Resin 20–3430128 (4 parts) and EpoxiCure 2 Hardener 20–3432128 (1 part) in silicon moulds and left to set for three days (Fig. S1b). After trialling several vertebral section widths (600, 450, 300, and 150μm), a Buehler IsoMet Low-Speed Diamond Blade Saw fitted with two 4-inch blades and a 3.5 inch 0.5mm plastic separator was used to cut one longitudinal section through the centre of each vertebral centra at a thickness of approximately 300–400μm (Fig. S1c). Staining the vertebral sections with 0.01% Crystal Violet solution (Schwartz 1983) as in Cuevas-Zimbrón et al. (2013) was trialled but did not substantially enhance banding clarity.

**Fig. 2:**
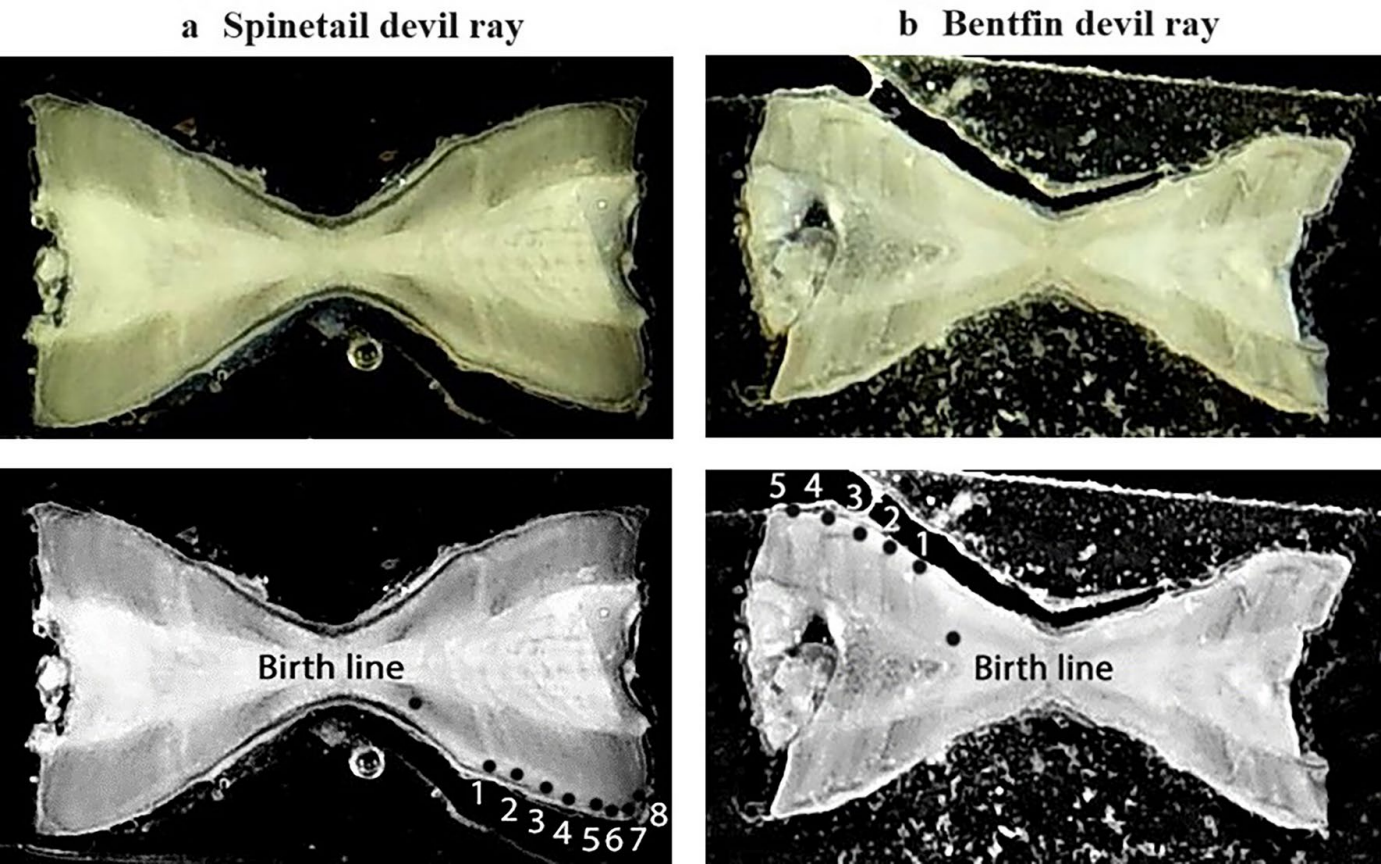
Images of **a**) Spinetail Devil Ray (*Mobula mobular*) and **b**) Bentfin Devil Ray (*M. thurstoni*) vertebrae sections for age determination using an Optika dissecting microscope with a fitted camera, illuminated from above using reflected light and from either side using a double-armed fibre optic light source, and enhanced in Adobe Photoshop Elements 2021 Photo Editor (Version: 19.0), annotated with birth line and annual growth bands. The imaged sections are from a) a male *M. mobular* aged as 8 years and b) a male *M. thurstoni* aged as 5 years caught in a small-scale gillnet fishery in Cilacap, Central Java, Indonesia between September–October 2020

A drop of water was added to the vertebral section, which was placed on black card prior to imaging. Each vertebral section was imaged using an Optika dissecting microscope with a fitted camera (Optika WF Series 4083.WiFi), illuminated from above using reflected light and from either side using a double-armed fibre optic light source (Fig. S1d). A 1.2X magnification was used for consistency. Optika Vision Lite 2.1 software was used to capture the image and export as a jpeg file. Each vertebral section was imaged on both sides and the image with the clearest view of the growth bands was used for age determination. Images of vertebral sections were enhanced in Adobe Photoshop Elements 2021 Photo Editor (Version: 19.0) following guidance in Campana (2014) to adjust the greyscale and sharpness to enhance the readability of banding patterns (Fig. 2).

Age was estimated based on the assumption of annual growth band pair deposition, with one translucent and one opaque band equating to one band pair indicating one year of age (Cailliet et al. 1983, 2006). The birth mark was identified as the first band with a distinct acute angle change in the *corpus calcareum*; each subsequent band pair was then assumed to represent one year of growth (Fig. 2) (Neer and Thompson 2005; Cuevas-Zimbrón et al. 2013; Campana 2014). Banding was read along the *corpus calcareum* near the lateral edge (Campana 2014). Adobe Photoshop Elements (2021 Photo Editor) was used to annotate enhanced images to indicate annual growth bands (Fig. 2). The bands were counted for each imaged section to age individuals to the nearest 0.5 year. A mean was then calculated from the counts of the two sections to give an estimated age per individual. Aging was conducted independently by two readers without access to contextual information such as animal size. If the mean estimates of the two readers differed by less than one year, the mean of these two values was taken as the best estimate for that individual (Goldman 2005). This is a more conservative approach than in many other studies due to the limited sample size (Smith et al. 2012; Temple et al. 2020). If mean estimates differed by more than one year, then ages were re-estimated with both readers together. If agreement was not reached (*n* = 0), samples would have been removed from further analyses (Goldman 2005).

The Bland–Altman approach for method comparison was used to quantify agreement, precision, and bias in age reads within each reader (comparing the age estimate between two sections per specimen) and between the two readers (comparing the mean age estimate for each specimen) (Bland and Altman 1999, 2003; Temple et al. 2020). Linear models of the mean age read for each specimen against the difference between reads for each specimen were used to check for bias in the relationship between reads (within and between readers). Limits of Agreement using the 95% mean confidence interval of the difference between reads was also used to define precision in age reads (within and between readers). Standard metrics of agreement used in aging studies were also presented for comparison: the average percent error, coefficient of variation, percent agreement and percent agreement ± 1 year (Beamish and Fournier 1981; Chang 1982) but note these latter measures are known to be flawed (Goldman 2005; Cailliet et al. 2006). Validation of band pair periodicity (i.e. the present assumption of annual growth band pair deposition) was not possible using marginal increment analysis or edge analysis due to insufficient sample numbers across all months of the year (Table S1 in the supplementary materials) and other validation methods such as mark-recapture of chemically tagged fish were not possible in this study (Campana 2001; Cailliet et al. 2006).

## Estimating growth

A Bayesian, multi-model approach was used to estimate growth using the DW-at-age dataset for *M. mobular* and *M. thurstoni*, incorporating prior knowledge of maximum size and size-at-birth to set informative priors (Pardo et al. 2016a). The DW-at-age datasets were missing samples for the largest individuals based on known maximum sizes and classical growth models using a frequentist approach are sensitive to missing data points (Siegfried and Sansó 2006). Therefore, a Bayesian approach likely provides growth estimates that are more biologically relevant than a classical, frequentist approach (Pardo et al. 2016a; Smart and Grammer 2021). Whilst the von Bertalanffy growth model is the most commonly used and generally best-fitting growth model for elasmobranchs, a multi-model approach is required to ensure the most appropriate model is fitted (Smart et al. 2016). We also fitted sigmoid functions (Gompertz and Logistic) as a common alternative that may suit taxa such as batoids due to their body shape as well as the Lester growth function as an example of a biphasic growth model (distinct juvenile and adult growth phases). An information-theoretic approach was used to choose the best fitting model by comparing leave-one-out information criterion (LOOIC) using the *loo* function from the R package *loo* (Vehtari et al. 2017, 2024). To account for multiplicative error, equations were log-transformed with an error term added.

The following models were fit and compared:

the three-parameter von Bertalanffy equation (von Bertalanffy, 1938):

1 $$log\left({DW}_{t}\right)= log\left({DW}_{\infty }-\left({{DW}_{\infty }- DW}_{0}\right){e}^{-kt}\right)+ {\in }_{t}$$

the Lester biphasic growth function (Lester et al. 2004):

2a $$log\left({DW}_{t}\right)=log\left(h\left(t-{t}_{1}\right)\right) + {\in }_{t}\,when\, t\le T$$

2b $$log\left({DW}_{t}\right)=log\left({DW}_{\infty }\left(1-{{e}^{-k \left(t- {DW}_{0}\right)}}\right)\right)+ {\in }_{t}\, when\, t>T$$

the three-parameter Gompertz growth function (Ricker 1975):

3 $$log\left({DW}_{t}\right)= log\left({DW}_{0}{e}^{\left(\text{log}\left(\frac{{DW}_{\infty }}{{DW}_{0}}\right)\left(1-{e}^{-gt}\right)\right)}\right) + {\in }_{t}$$

and the logistic growth function (Ricker 1979):

4 $$log\left({DW}_{t}\right)= log\left(\frac{{DW}_{\infty }{DW}_{0}{e}^{gt}}{{DW}_{\infty } + {DW}_{0}\left({e}^{gt} - 1\right)}\right)+ {\in }_{t}$$

where *DW*_*t*_ is the disc width at age *t*, *DW*_*∞*_ is the asymptotic disc width, *DW*_*0*_ is disc width at age zero,* k* and *g* are growth coefficients, *h* is the juvenile growth rate (disc width per unit time), *t*_*1*_ is the asymptotic hypothetical age at length 0, and *T* is the last immature age. The Lester biphasic growth model did not converge for either species and was therefore not reported in the results.

Reported maximum sizes are 350cm DW (individual from the Mediterranean) and 197cm DW (individual from the Philippines) for *M. mobular* and *M. thurstoni*, respectively, with size-at-birth reportedly 90–160cm and 70–90cm, respectively (Notarbartolo di Sciara 1988; Rambahiniarison et al. 2018; Notarbartolo di Sciara et al. 2020). Asymptotic size in fishes can be estimated from maximum size using the following equation (Froese and Binohlan 2000):

5 $${DW}_{\infty }={10}^{0.044+0.9841*{(\text{log}}_{10}{(DW}_{max}))}$$

where *DW*_*max*_ is the maximum size in centimetres. For a *DW*_*max*_ of 350cm for *M. mobular* and 197cm for *M. thurstoni*, this resulted in *DW*_*∞*_ = 1.01 * *DW*_*max*_ and *DW*_*∞*_ = 1.02 * *DW*_*max*_, respectively. Hyperpriors were set for this parameter, defined as *kappa,* and based on a *gamma* distribution with a mean of 1.01 and 1.02, respectively. The probability distribution of *kappa* was set between 0.7 and 1.3 (Froese and Binohlan 2000; Pardo et al. 2016a). Priors were also constrained for *DW*_*0*_ around size-at-birth. The same priors were used across models for DW_0_, DW_∞_, and σ for each species as these parameters can be interpreted in the same way (Smart et al. 2016; Smart and Grammer 2021) (Table 1). There is prior information on *k* for the von Bertalanffy growth model for *M. mobular* (Pardo et al. 2016a) but since the growth coefficient used is unique to each growth model tested and therefore not comparable across models (Smart et al. 2016), an uninformative prior was used for all models (Table 1). For the Lester biphasic growth model, *T* was constrained around the minimum age at maturity for *M. mobular*, which was estimated between five to six years in a previous study (Cuevas-Zimbrón et al. 2013; Pardo et al. 2016a). A prior with a normal distribution and a mean of four years was therefore used to account for a lag between the start of reproductive investment and maturity (Wilson et al. 2018). Age-at-maturity is unknown for *M. thurstoni* but is inferred from *M. mobular*. Uninformative priors were used for *h* and *t*_*1*_ parameters.

As well as setting informative priors, we compare the effect on posteriors with parameter estimates using weaker priors with the same mean of the distributions but higher variance (Table 1). A weakly informative prior is used for the variance σ^2^ in all growth models. We trialled fitting uninformative priors with uniform distributions but the model did not converge well because there were insufficient data to fit an asymptotic curve due to the low number of larger and older individuals; this means the chosen priors were not truly uninformative (Van Dongen 2006). Finally, we used the top model to test for potential regional differences in growth between Indian Ocean *M. mobular* (Indonesia and Pakistan) and *M. mobular* caught off Mexico (Cuevas-Zimbrón et al. 2013). Bayesian models were written in Stan and conducted in RStan version 2.21.0 (Stan Development Team 2023).

## Estimation of maximum intrinsic rate of population increase

The maximum intrinsic rate of population increase (*r*_*max*_) was estimated for *M. mobular* and *M. thurstoni* using a modified Euler-Lotka model that accounts for survival to maturity (Pardo et al. 2016b; Cortés 2016):

6 $${l}_{{\alpha }_{mat}}b= {e}^{{r}_{max}{\alpha }_{mat}}- {e}^{-M}({e}^{{r}_{max}}{)}^{{\alpha }_{{mat}^{-1}}}$$

where ${l}_{{\alpha }_{mat}}$ is survival to maturity, *b* is the annual reproductive output of female offspring, *α*_*mat*_ is female age-at-maturity, and *M* is the instantaneous rate of natural mortality. ${l}_{{\alpha }_{mat}}$ is calculated as:

7 $${l}_{{\alpha }_{mat}}=({e}^{-M}{)}^{{\alpha }_{mat}}$$

*M* is estimated as:

8 $$M= {\left(\frac{{\alpha }_{max}+ {\alpha }_{mat}}{2}\right)}^{-1}$$

where *α*_*max*_ is female maximum age. Note, this estimation of *r*_*max*_ is not comparable to estimates made using structured life table methods and Leslie matrix models, which rely on age- or stage-specific parameter estimates often lacking for chondrichthyans (Simpfendorfer 2005). The unstructured method of *r*_*max*_ estimation used here is therefore suitable for data-poor *M. mobular* and *M. thurstoni*. The limited sample size and temporal period of sampling in this study meant the annual reproductive output *b* of *M. mobular* and *M. thurstoni* could not be determined. Both species are known to produce a single pup, occasionally two pups per litter, over a 12-month gestation period and have a reproductive cycle of one to three years with resting periods (Doumbouya 2011; Rambahiniarison et al. 2018). Assuming a 1:1 sex ratio, we estimate a plausible range of *b* using the following equation:

9 $$b=0.5\left(\frac{l}{i}\right)$$

where *l* is litter size and* i* is breeding interval. *b* was therefore bound between 0.17 (based on a single pup and triennial reproductive cycle) and 1 (two pups and annual reproductive cycle). *α*_*mat*_ and DW at 50% maturity *DW*_*mat*_ were estimated using Bayesian logistic regressions for both species for combined sex using strong and weaker priors. However, *M. thurstoni* logistic regression models did not fit the DW- and age-maturity data well due to limited observations of mature individuals (*n* = 2) and therefore parameter estimates are not presented. Parameter estimates for *M. mobular* were similar and therefore only presented for stronger priors in the results. There are no direct estimates of age at maturity available for *M. thurstoni* but there is an estimate of *DW*_*mat*_ of 150cm (Notarbartolo di Sciara 1988; Rambahiniarison et al. 2018). Using age and growth data from this study, *α*_*mat*_ for *M. thurstoni* was therefore assumed to mature between five and six years. Size at maturity for females and males of both species have been found to be similar (Stevens et al. 2018; Rambahiniarison et al. 2018). We used the range of *α*_*mat*_ estimated to set the lower and upper bounds of *α*_*mat*_ for each species.

To estimate *M*, we need to estimate *α*_*max*_. We used the theoretical age that each species reached 95% and 99% DW_∞_ to estimate *α*_*max*_ for the von Bertalanffy and logistic functions, respectively. The former is calculated as 5ln(2)*k*^−1^ (Ricker 1979) and the latter (Smart et al. 2016; Grant et al. 2020), as:

10 $$\raisebox{1ex}{$log\left\{\frac{0.99{L}_{\infty }\times {L}_{\infty }\times {L}_{0}}{{L}_{0}[{L}_{\infty }-{0.99L}_{\infty }]} \right\}$}\!\left/ \!\raisebox{-1ex}{$g$}\right.$$

99% DW_∞_ was used for the logistic function because the asymptote is reached at younger ages than the von Bertalanffy and therefore provides a more realistic estimate of longevity. The maximum observed age in this study was less than the 95% DW_∞_ and was therefore used as the lower bound. *r*_max_ was estimated using the *nlminb* function in R from the package *stats* (R Core Team 2021). A Monte Carlo approach was used whereby 10,000 random deviates were drawn from a uniform distribution between minimum and maximum values of *b*, *α*_*mat*_, and *α*_*max*_ to account for uncertainty within these parameters (Dulvy et al. 2014; Temple et al. 2020). The same approach was used to estimate *M* from a triangular distribution using the 95% quantiles (as estimated using the 10,000 random deviates of *α*_*mat*_ and *α*_*max*_).

## Estimation of total mortality, fishing mortality, and the exploitation ratio

The top growth model was used to estimate age to the nearest year for *M. mobular* (*n* = 103) and *M. thurstoni* (*n* = 89) given DW. Total instantaneous mortality *Z* (± 95% CI), which is a combination of fishing mortality *F* and natural mortality *M*, was calculated using the Chapman-Robson catch curve with the package *FSA* (Smith et al. 2012). This assumes the individuals in this study are one population with minimal migration and that sampling from the fishery is random and non-selective across age and size classes. The ages of *M. mobular* and *M. thurstoni* fully recruited to the fishery (three and two years, respectively) were assumed to be the peak abundance, with *Z* estimated from four and three years of age, respectively. 10,000 draws were made from the estimated ranges of *M* and *Z,* with uniform and normal probability distributions assumed, respectively. *F* was estimated by subtracting the ranges of natural mortality *M* from the ranges of *Z*. Exploitation ratio *E* was calculated by dividing the ranges of fishing mortality *F* by the ranges of *Z*. Median *F*, *r*_max_, and exploitation ratio *E* estimates were compared whereby *r*_*max*_ is equivalent to the fishing mortality that will drive a species to extinction (*F*_*extinc*t_) and *E* is the ratio of *F* to *M* and if *F* = *M* then* E* is 0.5, representing an optimum value for biological sustainability (Gulland 1971; Pauly 1983).

All data analyses and visualisations were conducted in R version 4.1.2 (R Core Team 2021).

## Results

### Disc width-weight relationship

*Mobula mobular* sampled in this study ranged between 62 and 260 cm DW, whilst *M. thurstoni* ranged between 75 and 190 cm DW (Fig. S2 in the supplementary materials). A higher number (*n* = 51, 57% of total number) of female *M. thurstoni* were sampled compared to males (*n* = 38) whilst a similar number of female (*n* = 52) and male (*n* = 51) *M. mobular* were sampled. Juveniles and mature individuals of both sexes were sampled for each species as indicated by maturity status assessments as well as known offspring size and size at maturity (IUCN, 2022) (Fig. S2). However, only two mature *M. thurstoni* individuals were recorded (both male). Two individuals of *M. mobular* were smaller (62 cm and 87 cm DW) than the minimum known offspring size for this species (90 cm DW) (Fig. S2). A few individuals close to the known maximum size of *M. thurstoni* were sampled; however, this species possibly reaches 220 cm DW (Jabado and Ebert 2015) and the largest possible individuals of *M. mobular* were likely not sampled as indicated by known maximum sizes (IUCN, 2022) (Fig. S2 in the supplementary materials).

**Fig. 3:**
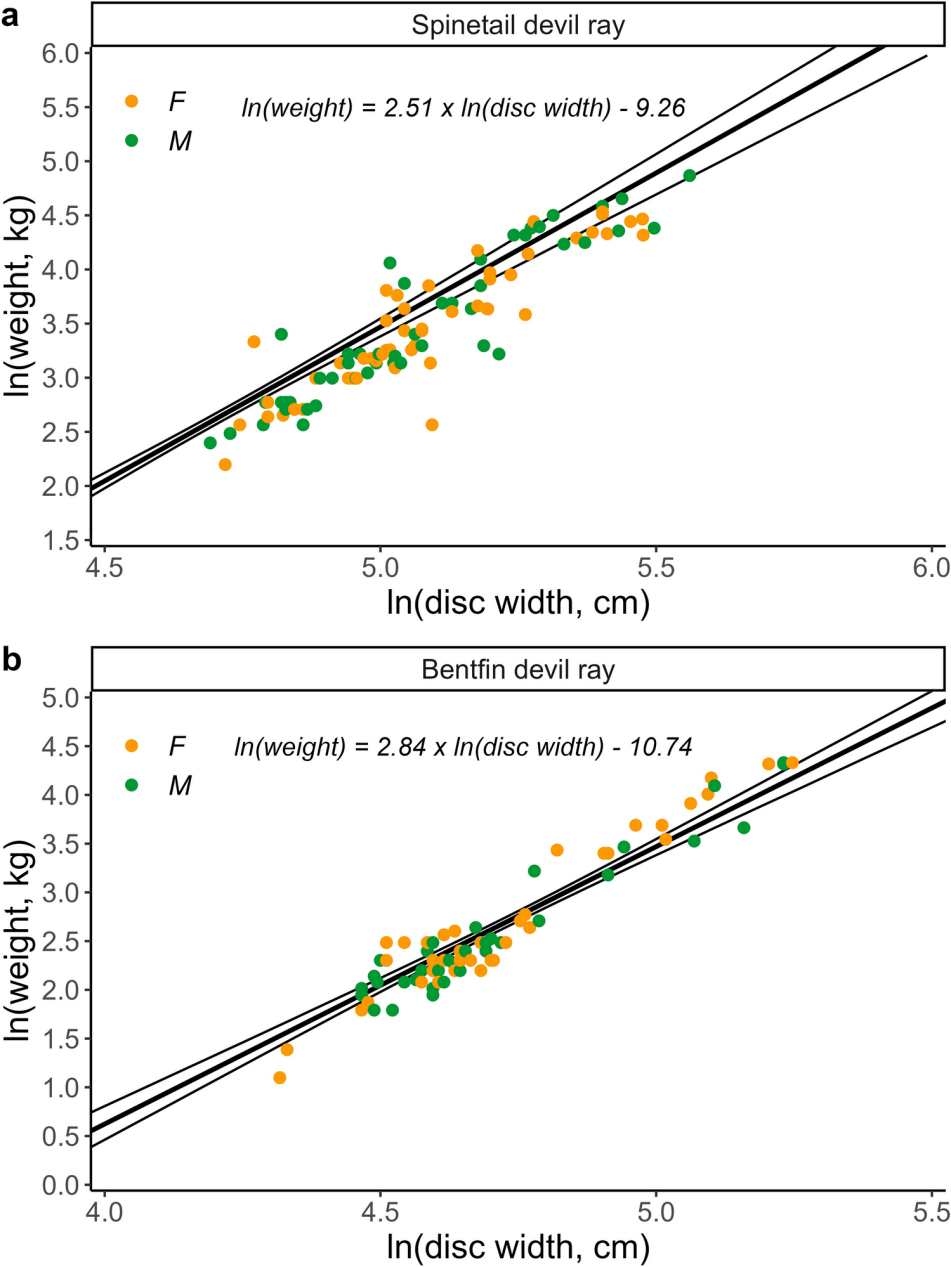
Natural log-transformed disc width (cm) and weight (kg) Bayesian linear relationship (with 95% credible intervals) for female (orange) and male (green) **a**) Spinetail Devil Ray (*M. mobular*) (*n* = 101) and **b**) Bentfin Devil Ray (*M. thurstoni*) (*n* = 76)

There was little difference in *a* and *b* estimates with strong and weaker priors and therefore the Bayesian linear disc width-weight relationship was presented for strong priors only for each species (Table 2; Fig. 3).

**Table 2:** Mean estimates (95% Credible Intervals) of Bayesian length–weight regression, growth models, and length-maturity regression models using strong and weaker priors for Spinetail Devil Ray (*Mobula mobular*) (*n* = 79) and Bentfin Devil Ray (*M. thurstoni*) (*n* = 59)

Species	Model	Parameter	Strong priors	Weaker priors
*Mobula mobular*	DW-weight linear regression	*b*	2.51 (2.30, 2.71)	2.51 (2.31, 2.70)
		*log(a)*	−9.26 (− 10.28, − 8.19)	−9.27 (− 10.25, − 8.24)
		σ^2^	0.27 (0.24, 0.32)	0.27 (0.24, 0.32)
	von Bertalanffy growth model	*k*	0.05 (0.04, 0.06)	0.06 (0.03, 0.11)
		*DW*_*0*_	1166.53 (1085.5, 1246.79)	1162.64 (1070.34, 1249.08)
		*DW*_*∞*_	3502.95 (3214.2, 3795.37)	3311.95 (2545.35, 4184.92)
		σ^2^	0.12 (0.1, 0.14)	0.12 (0.1, 0.14)
	Gompertz growth model	*g*	0.08 (0.06, 0.09)	0.09 (0.06, 0.16)
		*DW*_*0*_	1197.67 (1125.27, 1270.53)	1186.47 (1096.77, 1269)
		*DW*_*∞*_	3490.26 (3195.5, 3789.21)	3146.85 (2439.22, 4073.06)
		σ^2^	0.12 (0.1, 0.14)	0.12 (0.1, 0.14)
	Logistic growth model	*g*	0.11 (0.08, 0.13)	0.14 (0.09, 0.22)
		*DW*_*0*_	1222.28 (1158.27, 1287.63)	1200.65 (1111.93, 1284.85)
		*DW*_*∞*_	3474.85 (3187, 3773.44)	2994.05 (2340.35, 3938.02)
		σ^2^	0.12 (0.1, 0.14)	0.12 (0.1, 0.14)
	Age-maturity logistic regression	*β*	0.66 (0.35, 1.04)	0.70 (0.37, 1.09)
		*a*	−5.46 (− 8.49, − 3.24)	−5.70 (− 8.74, − 3.32)
	DW-maturity logistic regression	*β*	0.07 (0.04, 0.09)	0.10 (0.06, 0.16)
		*a*	−13.72 (− 19.0, − 8.90)	−20.79 (− 31.62, − 12.71)
*Mobula thurstoni*	DW-weight linear regression	*β*	2.84 (2.60, 3.06)	2.84 (2.61, 3.06)
		*log(a)*	−10.74 (− 11.77, − 9.61)	−10.74 (− 11.79, − 9.63)
			0.23 (0.19, 0.28)	0.23 (0.19, 0.28)
	von Bertalanffy growth model	*k*	0.1 (0.07, 0.15)	0.11 (0.05, 0.24)
		*DW*_*0*_	849.86 (789.17, 906.01)	851.93 (788.04, 907.96)
		*DW*_*∞*_	2014.78 (1777.36, 2245.42)	2113.41 (1481.71, 2845.3)
		σ^2^	0.1 (0.08, 0.12)	0.1 (0.08, 0.12)
	Gompertz growth model	*g*	0.14 (0.09, 0.22)	0.14 (0.08, 0.26)
		*DW*_*0*_	862.72 (811.98, 911.67)	864.77 (809.56, 915.81)
		*DW*_*∞*_	2047.37 (1664.56, 2438.92)	2155.38 (1524.94, 2909.55)
		σ^2^	0.1 (0.08, 0.12)	0.1 (0.08, 0.12)
	Logistic growth model	*g*	0.19 (0.14, 0.25)	0.18 (0.12, 0.31)
		*DW*_*0*_	870.21 (823.47, 916.42)	873 (821.75, 920.05)
		*DW*_*∞*_	2022.11 (1796.58, 2252.17)	2178.12 (1549.54, 2929.75)
		σ^2^	0.1 (0.08, 0.12)	0.09 (0.08, 0.11)

## Age estimation using caudal vertebrae

Vertebral samples for age estimation in *M. mobular* (*n* = 79) came from Indonesia (*n* = 48) and Pakistan (*n* = 31), with individuals younger than one years old sampled for both sex and maximum ages of 17.5 and 12.5 years for females (*n* = 41) and males (*n* = 38), respectively. Vertebral samples for age estimation in *M. thurstoni* (*n* = 59) came from Indonesia, with all females (*n* = 29) aged under two years old and males (*n* = 30) ranging from less than one years old to six years old. The caudal vertebrae of *M. tarapacana* (*n* = 3) showed clear banding, suggesting this method would be viable for future aging studies with a greater sample size. The three individuals sampled were aged at 1.5, 6, and 8 years for specimens with 152 (female), 237 (male), and 210 cm (female) DW, respectively.

Bland–Altman analyses of *M. mobular* reads showed no evidence of bias within reader 1 (*M. mobular*, *R*^*2*^ = 0.0259, *F* = 3.08, *P* = 0.0834; *M. thurstoni*, *R*^*2*^ = 0.0170, *F* = 2.00, *P* = 0.163) or reader 2 (*M. mobular*, *R*^*2*^ = 0.00761, *F* = 1.60, *P* = 0.210; *M. thurstoni*, *R*^*2*^ = 0.00504, *F* = 1.29, *P* = 0.260) (Fig. S3 in the supplementary materials). There was evidence of significant bias between readers for *M. mobular* (*R*^*2*^ = 0.0742, *F* = 7.26, *P* < 0.001) but not for *M. thurstoni* (*R*^*2*^ = 0.0126, *F* = 1.74, *P* = 0.192) (Fig. S3). For all individuals that initially differed by more than one year, consensus was reached between readers, likely overcoming this bias. Average percent error, coefficient of variation, percent agreement, percent agreement ± 1 year are also presented alongside Bland–Altman limits of agreement (Table 3). The variability in age band counts was consistent with other shark and ray aging studies (Jacobsen and Bennett 2010; Gutteridge et al. 2013; Baje et al. 2018; Temple et al. 2020). Higher variability in *M. thurstoni* reads is likely due to younger age estimates meaning smaller differences in age band counts can cause inflated error estimates (Baje et al. 2018).

**Table 3:** Estimates of ageing agreement, precision, and bias in age reads for Spinetail Devil Ray (*Mobula mobular*) (*n* = 79) and Bentfin devil ray (*M. thurstoni*) (*n* = 59) within and between two readers: Average Percent Error (APE), Coefficient of Variation (CV), Percent Agreement (PA), PA ± 1 year, and Bland–Altman Limits of Agreement (LOA)

Species	Estimate	PA (%)	PA ± 1 year (%)	CV (%)	APE (%)	LOA (± years)
*Mobula mobular*	Within reader 1	64.6	23.3	5.94	4.20	1.63
	Within reader 2	36.7	50.7	15.8	11.2	2.03
	Between readers 1 and 2	41.8	22.7	10.1	7.17	1.44
*Mobula thurstoni*	Within reader 1	81.4	5.88	3.86	2.73	0.57
	Within reader 2	89.8	7.02	2.52	1.78	0.54
	Between readers 1 and 2	78.0	0.00	2.44	1.73	0.33

## Estimating growth

**Fig. 4:**
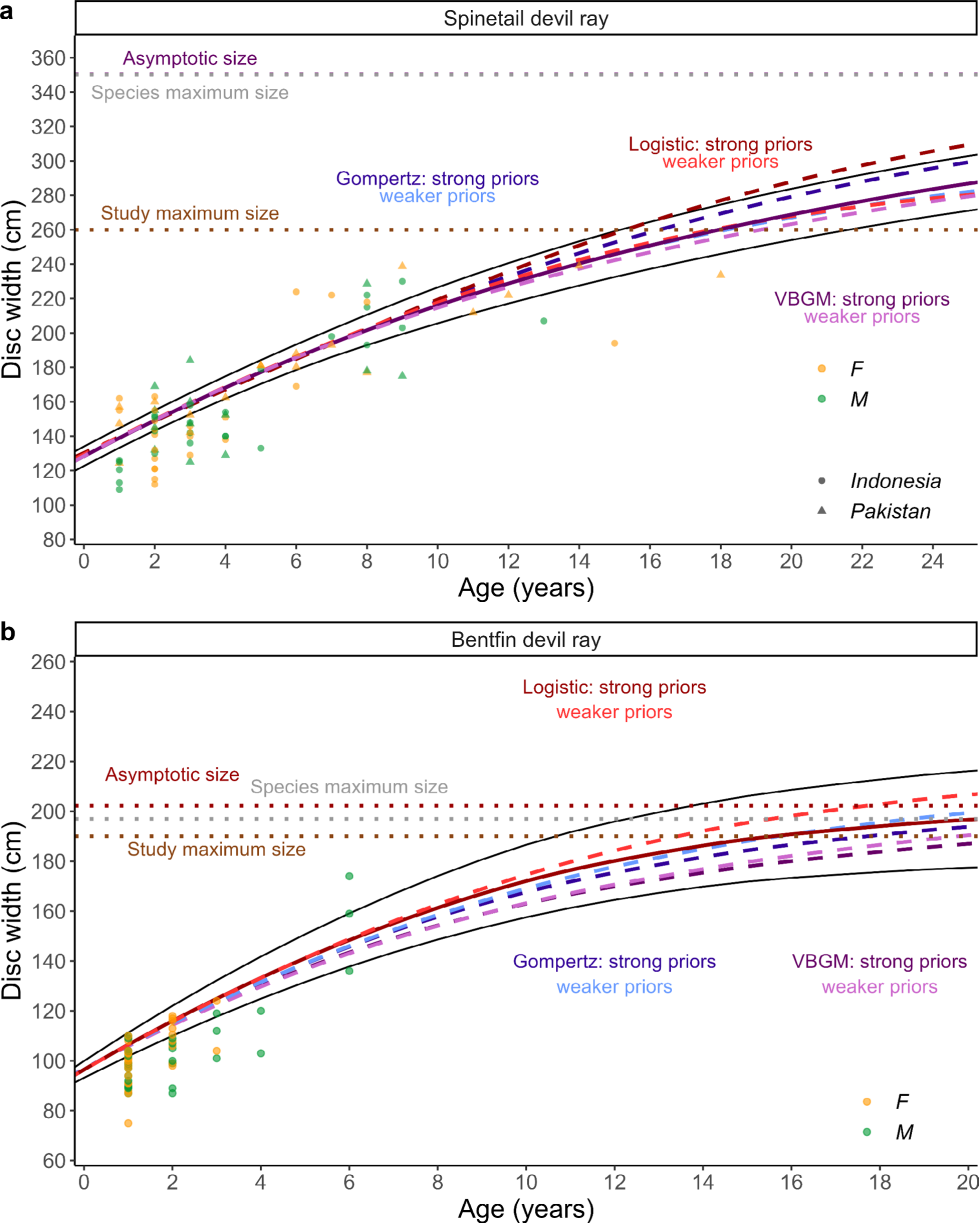
Bayesian von Bertalanffy (purples), Gompertz (blues), and logistic (reds) growth curves describing the disc width and age (nearest year) relationship for a) combined female (*n* = 41) and male (*n* = 38) Spinetail Devil Ray (*M. mobular*) (*n* = 79) and b) combined female (*n* = 29) and male (*n* = 30) Bentfin Devil Ray (*M. thurstoni*) (*n* = 59) length-at-age data from individuals sampled in Indonesia (circles) and Pakistan (triangles). Top model for each species shown with a solid line and remaining models with dashed lines. Dotted lines show the asymptotic size (*DW*_*∞*_) estimate for the top model, the maximum observed size for the species (black), and the maximum observed size in this study (brown)

**Fig. 5:**
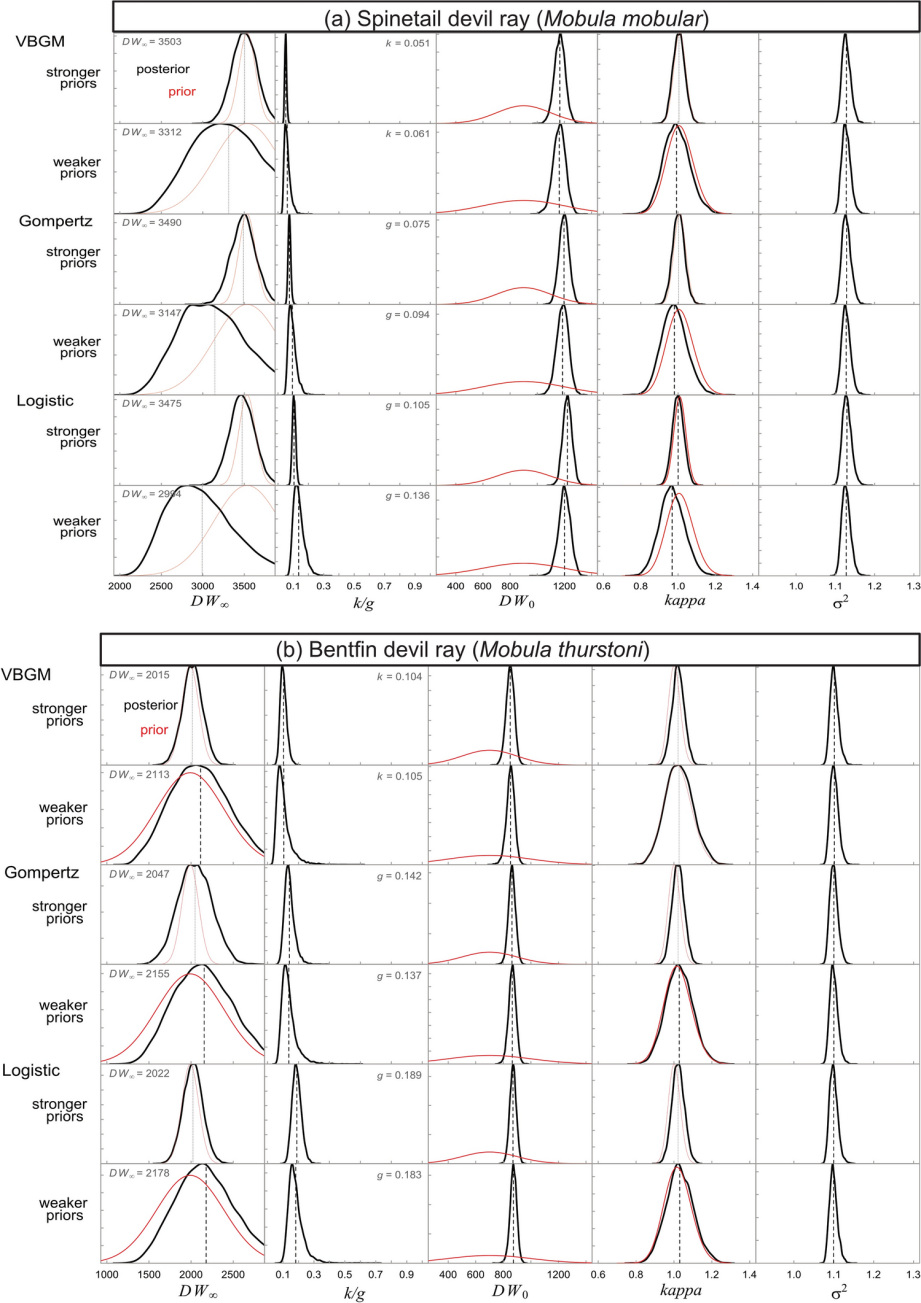
Posterior (black lines) and prior (red lines) distributions for von Bertalanffy growth parameters (*k/g*, *DW*_*∞*_, and *DW*_*0*_), the hyperprior *kappa*, and the error term (σ^2^) for three Bayesian models (von Bertalanffy, Gompertz, and Logistic) with strong and weaker priors fitted to a) Spinetail Devil Ray (*Mobula mobular*) and b) Bentfin Devil Ray (*M. thurstoni*) disc width-at-age data. Dashed lines show mean values and mean growth coefficient and *DW*_*∞*_, indicated on each plot

**Fig. 6:**
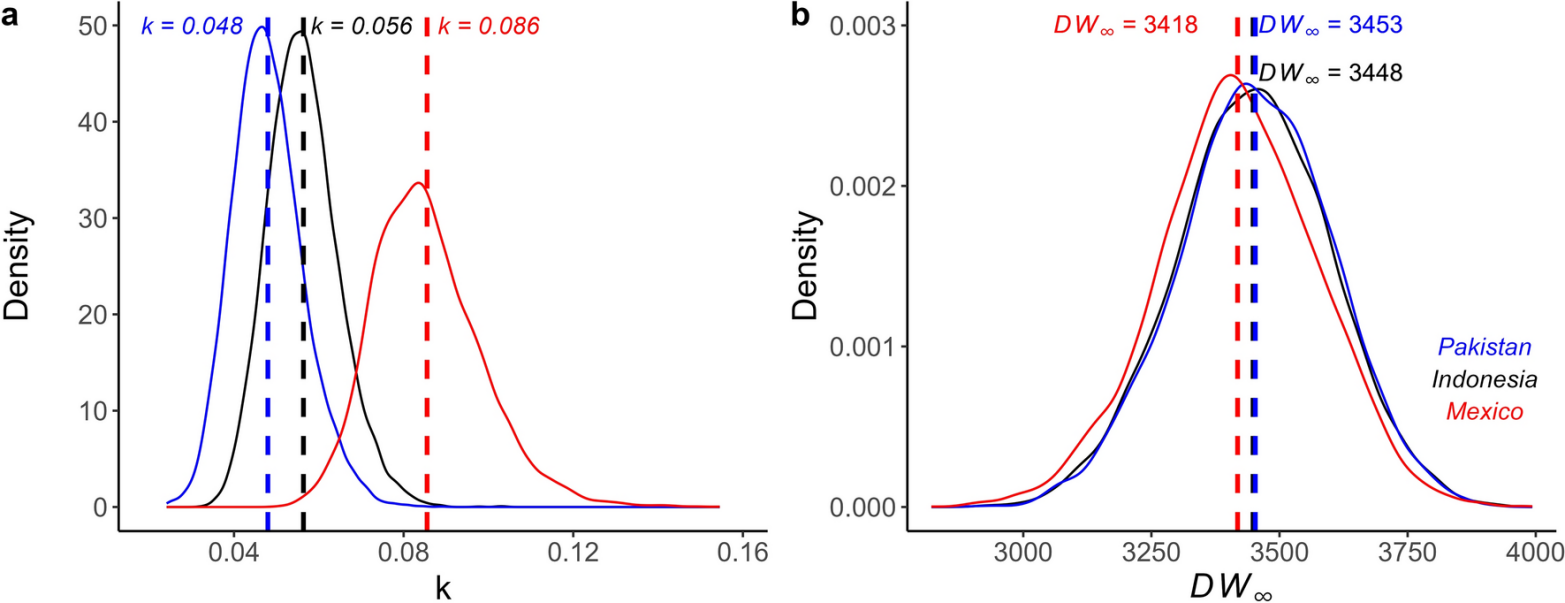
Posterior distribution for von Bertalanffy growth parameters **a**) *k* and **b**) *DW*_*∞*_ for Bayesian models with strong priors fitted to Indian Ocean Spinetail Devil Ray (*M. mobular*) disc width-at-age data from this study using samples from Indonesia (black) and Pakistan (blue) and previous studies using samples from Mexico (red) (Cuevas-Zimbrón et al. 2013; Pardo et al. 2016a)

Of the four growth models tested, the three-parameter von Bertalanffy and logistic growth models with stronger priors fit best for *M. mobular* and *M. thurstoni* DW-at-age data, respectively, based on LOOIC (Table 4; Fig. 4). These top models resulted in growth coefficients and *DW*_*∞*_ estimates of *k* = 0.05 year^−1^ and 350cm for *M. mobular* and *g* = 0.19 year^−1^ and 202cm for *M. thurstoni*, respectively. The mean *DW*_*∞*_ estimates from the top models were in line with maximum observed sizes for *M. mobular* (350cm) and *M. thurstoni* (197cm), suggesting the Bayesian models produced plausible estimates of growth coefficients. Bayesian models with strong priors resulted in lower mean *k* estimates and higher mean *DW*_*∞*_ estimates for *M. mobular* compared to models with weaker priors (Table 2; Fig. 5). Whereas, models with weaker priors resulted in higher or the same mean *g* and *DW*_*∞*_ estimates for *M. thurstoni* (Table 2; Fig. 5). The *M. mobular k* estimate from the top model was lower than a previous estimate that also fitted a von Bertalanffy growth model (0.12 year^−1^) (Pardo et al. 2016a) to the only other published length-at-age dataset for this species sampled in Mexico (Cuevas-Zimbrón et al. 2013) (Fig. 6). We used a different offspring and maximum size as informative priors (based on currently available literature) to re-estimate* k* for the Mexico (0.086 year^−1^) dataset as well as for *M. mobular* sampled in Indonesia (0.056 year^−1^) and Pakistan (0.048 year^−1^) in this study; this also showed Indian Ocean devil rays had lower growth coefficients (Fig. 6). Parameter estimates from all growth models are presented (Table 2).

**Table 4:** ‘Leave One Out’ cross validation Information Criterion (LOOIC ± standard error, se) for growth model analyses for Spinetail Devil Ray (*Mobula mobular*) and Bentfin Devil Ray (*M. thurstoni*). The best model with the lowest LOOIC and largest weight for each species is shown in bold

Species	Model	Parameter	LOOIC	LOOIC se	Weight
*Mobula mobular*	**Von Bertalanffy**	**Strong**	**−109.11**	**10.04**	**1**
	Von Bertalanffy	Weaker	−108.89	10.11	3.62E−06
	Gompertz	Strong	−108.31	10.01	6.39E−07
	Gompertz	Weaker	−108.96	10.04	3.24E−05
	Logistic	Strong	−106.97	10.03	7.95E−07
	Logistic	Weaker	−108.74	10.02	2.42E−05
*Mobula thurstoni*	Von Bertalanffy	Strong	−106.06	11.98	2.49E−09
	Von Bertalanffy	Weaker	−105.97	12.11	4.54E−07
	Gompertz	Strong	−107.28	11.87	1.80E−06
	Gompertz	Weaker	−107.00	11.98	3.23E−08
	**Logistic**	**Strong**	**−108.19**	**11.59**	**1**
	Logistic	Weaker	−108.01	11.83	0.000406

## Estimation of maximum intrinsic rate of population increase

**Fig. 7:**
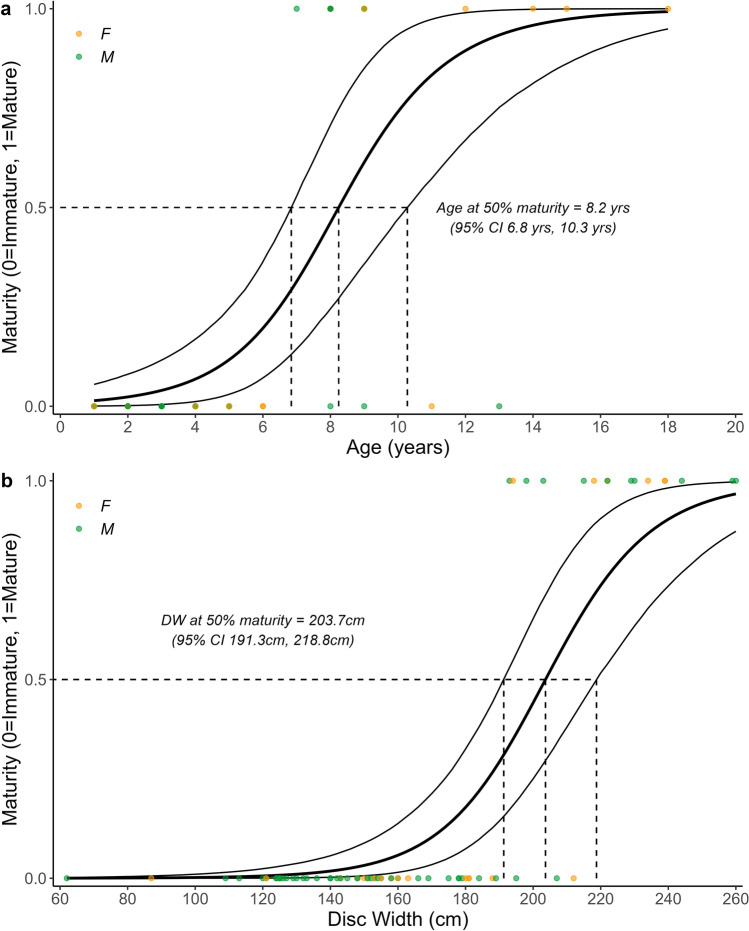
Bayesian logistic regression with strong priors describing the relationship between **a**) age (*n* = 56) and **b**) disc width (*n* = 73) and maturity status for Spinetail Devil Ray (*M. mobular*) with 95% Credible Intervals. Age and disc width at 50% maturity (with 95% Credible Intervals) are shown with dashed lines

*M. mobular* had an estimated mean *α*_*mat*_ of 8.2 years (95% CI 6.8 years, 10.3 years) and mean *DW*_*mat*_ of 204cm (95% CI 191cm, 219cm) (Fig. 7). The smallest observed mature *M. mobular* and *M. thurstoni* individuals were 193 and 171cm, which equates to 7.9 and 10.4 years, respectively (predicted using the top fitting growth model for each species). The former is within the calculated range of age at maturity for *M. mobular* and we therefore assume female *α*_*mat*_ ranges uniformly between 6.8 and 10.3 years. For *M. thurstoni*, a *DW*_*mat*_ of 150cm from a previous study (Notarbartolo di Sciara 1988; Rambahiniarison et al. 2018) equates to 7 years and we therefore assume *α*_*mat*_ ranges uniformly between 7 and 10.4 years.

**Fig. 8:**
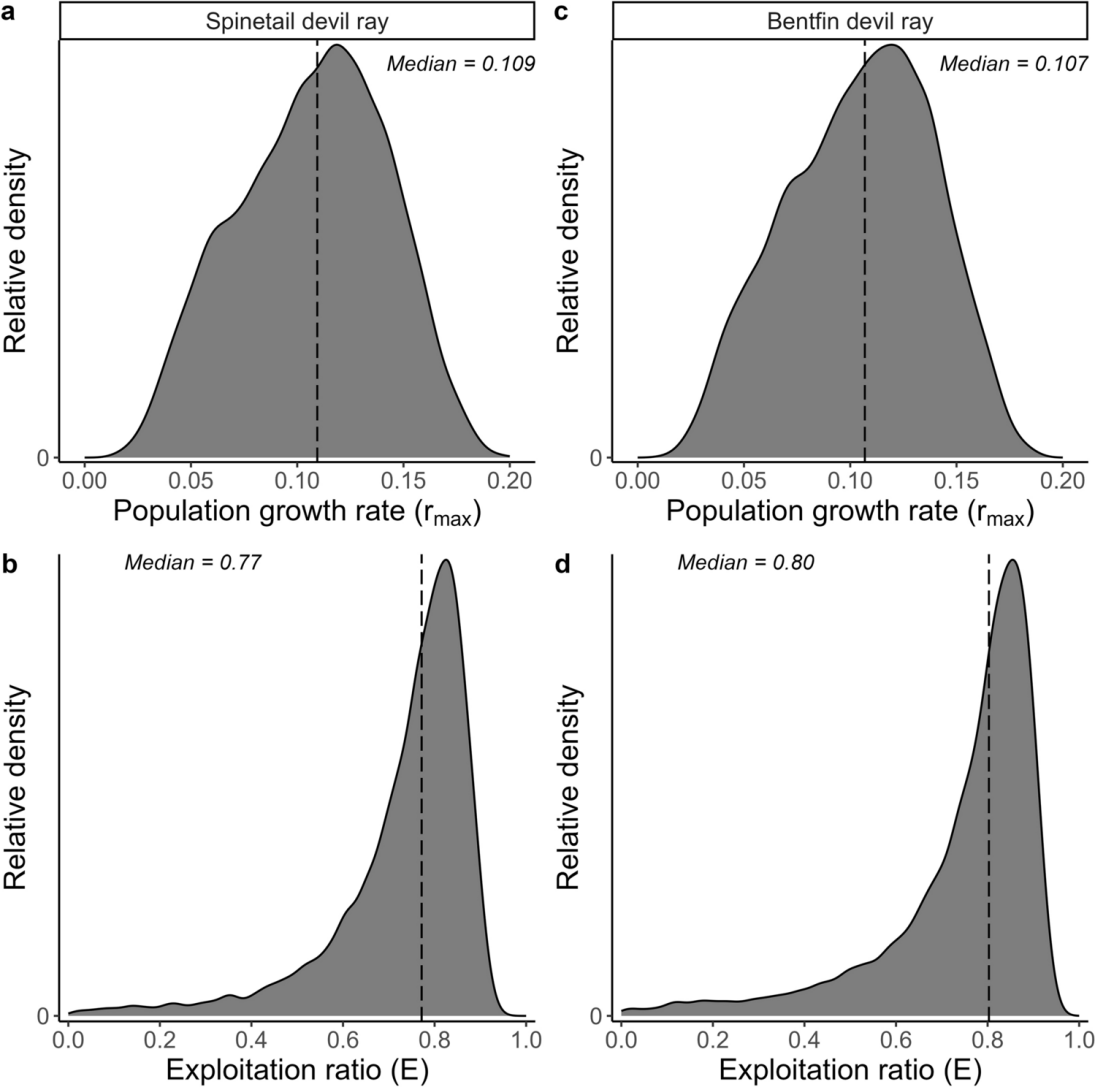
Distribution of estimated maximum intrinsic population growth rate (r_max_) and exploitation ratio (*E*) for Spinetail Devil Ray (*M. mobular*) (**a** and **b**, respectively) and Bentfin Devil Ray (*M. thurstoni*) (**c** and **d**, respectively). Dashed lines show median values

Female *α*_max_ was calculated based on age at 95% *DW*_∞_ (Ricker 1979) and 99% *DW*_∞_ (Smart et al. 2016; Grant et al. 2020) giving 69.3 and 64.3 years for *M. mobular* and *M. thurstoni*, respectively. These *α*_max_ estimates are likely unrealistically high due to the growth curves not reaching an asymptote. The maximum observed age of *M. thurstoni* was six years but this is unlikely to represent true maximum age. The observed maximum age of *M. mobular* in this study was 17.5 years and so this was used as the lower bound for *α*_max_ for both species. Therefore *α*_max_ was assumed to uniformly range between 17.5 and 69 years and 17.5 and 64 years for *M. mobular* and *M. thurstoni*, respectively. Using the 10,000 drawn estimates of *b*, *α*_mat_, and *α*_max_, median instantaneous natural mortality *M* was calculated as 0.055 (95th percentiles 0.033, 0.070) for *M. mobular* and 0.057 (95th percentiles 0.034, 0.070) for *M. thurstoni*. Resultant median *r*_max_ was calculated as 0.109 year^−1^ (95th percentiles 0.039, 0.168) for *M. mobular* and 0.107 year^−1^ (95th percentiles 0.038, 0.164) for *M. thurstoni*, respectively (Fig. 8). We trialled reducing the upper bound of *α*_max_ to see how it affected *r*_max_ estimations e.g. to 26 years and median *r*_max_ values were only slightly reduced (from 0.0107 yr^−1^ to 0.091 yr^−1^).

## Estimation of total mortality, fishing mortality, and the exploitation ratio

**Fig. 9:**
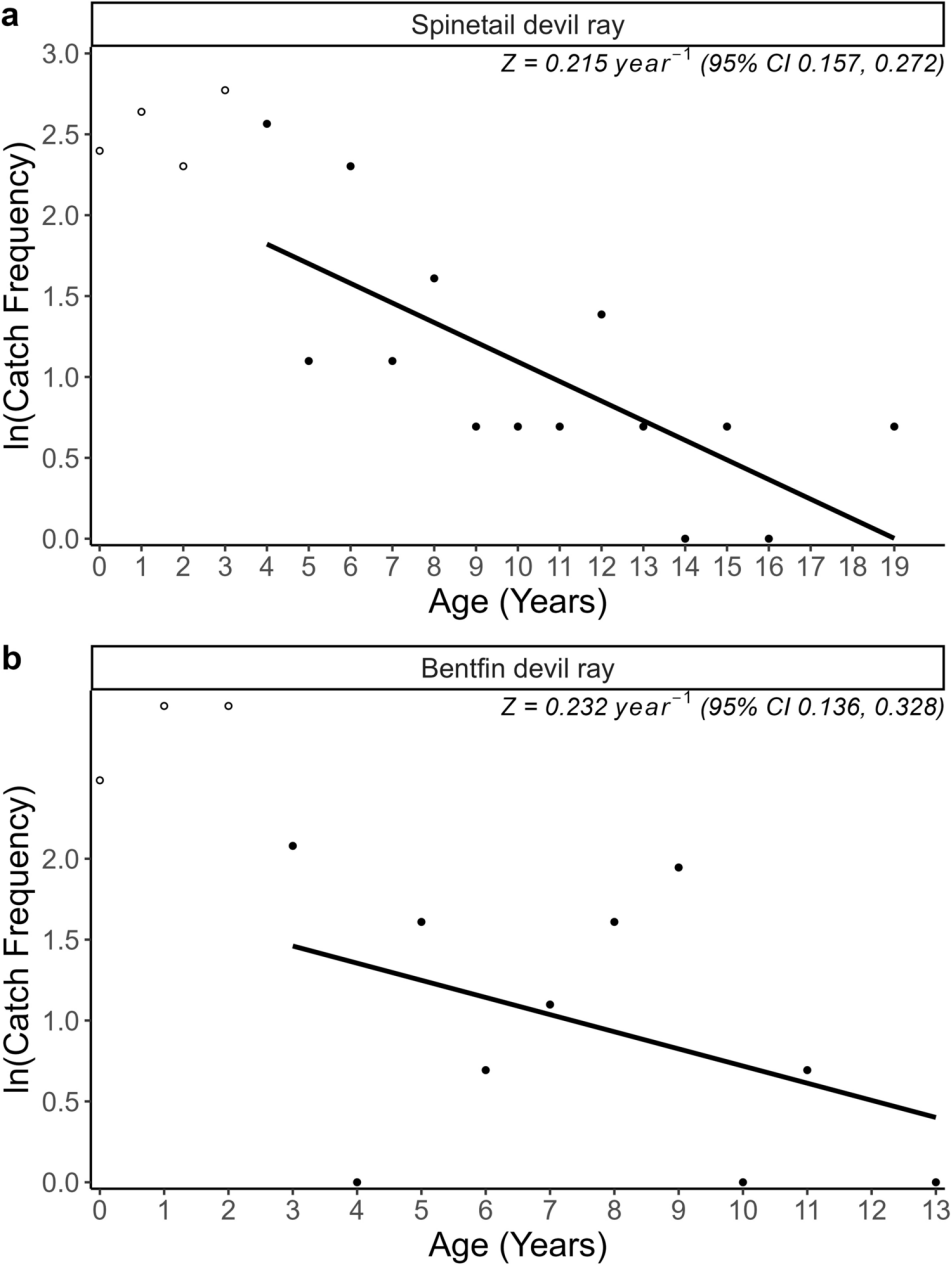
Chapman-Robson catch curve for **a**) Spinetail Devil Ray (*Mobula mobular*) (*n* = 103) and **b**) Bentfin Devil Ray (*M. thurstoni*) (*n* = 89) from Indian Ocean small-scale fisheries. Age class for full recruitment to the fishery was 3 and 2 years, respectively, and catch curve regression lines between ages 4 to 18 and 3 to 13, respectively. Total mortality *Z* indicated

Full recruitment to the fishery for *M. mobular* and *M. thurstoni* was estimated at three and two years, respectively, based on the peak abundance from catch curve analysis (Fig. 9). Total mortality *Z* was similar for both species (*M. mobular*: 0.215 year^−1^, 95% CI 0.157, 0.272; *M. thurstoni:* 0.232 year^−1^, 95% CI 0.136, 0.328), which translated to a total annual mortality rate of approximately 20% (*M. mobular*: 19.3%, 95% CI 0.146, 0.238; *M. thurstoni*: 20.7%, 95% CI 0.128, 0.280). Median fishing mortality *F* was estimated as 0.16 year^−1^ (95th percentiles − 0.029, 0.358) and 0.18 year^−1^ (95th percentiles − 0.117, 0.472) for *M. mobular* and *M. thurstoni*, respectively. Estimates of *F* for both species, although highly uncertain, are higher than our *r*_*max*_ estimates (0.109 and 0.107 year^−1^), suggesting that current fishing mortality will drive the species towards extinction and is therefore unsustainable, within the assumptions made (Myers and Mertz 1998; Dulvy et al. 2004; Gedamke et al. 2007). The majority of sampled individuals were caught in gillnets, which are likely unselective for the size range of devil rays. However, the largest individuals were not sampled, likely because they are more difficult to catch meaning selectivity and therefore total mortality *Z* decreases with larger disc width leading to lower fishing mortality *F* for any given natural mortality *M* estimate (*Z* – *M* = *F*). Estimated median exploitation ratio *E* (ratio of *F* to *M*) was estimated as 0.77 (95th percentiles − 0.19, 0.91) for *M. mobular* and 0.80 (95th percentiles − 0.77, 2.38) for *M. thurstoni* (Fig. 8). Approximately 89% and 86% of the proportion of the estimated distribution of *E* is greater than the optimal value for biological sustainability of *E* = 0.5 for *M. mobular* and *M. thurstoni*, respectively, reinforcing that there is a high likelihood that *M. mobular* and *M. thurstoni* are being overfished (Fig. 8) (Gulland 1971; Pauly 1983).

## Discussion

Our results indicate that current fisheries exploitation of devil rays in Indian Ocean small-scale fisheries is unsustainable, with fishing mortality higher than *r*_*max*_ estimates and exploitation ratio exceeding a threshold for biological sustainability. We found that both *M. mobular* and *M. thurstoni* had low somatic and population growth rates (low *r*_*max*_), relative to most other chondrichthyans. Indian Ocean *M. mobular* also had a slower growth than found for this species in another region. We present the first published age and growth estimates for *M. thurstoni*, the first direct age-at-maturity estimate for any *Mobula* species, and only the second published aging study for *M. mobular,* including a record of the oldest individual published. In the following, we discuss (1) unsustainable fisheries catches of Indian Ocean devil rays; (2) how life history estimates compare to these species in other regions; (3) regional and global management implications; and (4) future research directions and caveats.

The distribution of the exploitation ratio *E* for both *M. mobular* and *M. thurstoni*, alongside the disparity between fishing mortality *F* and *r*_*max*_, suggests a high likelihood of overfishing. We found that *M. mobular* (*r*_*max*_ = 0.109 year^−1^) and *M. thurstoni* (*r*_*max*_ = 0.107 year^−1^) had low *r*_*max*_, which aligns with other studies that found devil rays have amongst the lowest *r*_*max*_ of all chondrichthyans, alongside deep sea species (Simpfendorfer and Kyne 2009; Dulvy et al. 2014; Pardo et al. 2016a). This is likely due to very low reproductive outputs (Pardo et al. 2018). Although we did not observe any pregnant females in our study, a litter size of one, rarely two pups, has been observed in several studies for *M. mobular* and other devil ray species (Notarbartolo di Sciara 1988; Broadhurst et al. 2018; Rambahiniarison et al. 2018). The low fecundity and large offspring sizes of devil rays indicates a life history strategy of high maternal investment and consequently lower juvenile natural mortality. This means that these species likely have weaker density-dependent regulation due to limited plasticity in life history and therefore lower potential to withstand and recover from additional fishing mortality (Forrest and Walters 2009; Kindsvater et al. 2016). That is not to say that high fecundity alone is indicative of greater resilience (Reynolds et al. 2005; Kindsvater et al. 2016). We used an age-independent estimation of natural mortality *M*, which retains comparability across studies that have commonly used this method for estimation of *r*_*max*_ in elasmobranchs where age-specific natural mortality is rarely known. The lack of older individuals observed led to uncertainty in maximum age estimates, which had to be estimated from the growth curves that were slow to asymptote (due to a lack of older individuals). This larger range in *α*_*max*_ better accounts for the uncertainty in *M* (due to subsequently larger range). Further, reducing our range of *α*_*max*_ estimates only marginally reduced *r*_*max*_ estimates. However, it is still important to acknowledge that* M* likely varies across size classes and comparison with age-dependent *M* estimators and other methods more broadly would be beneficial to assess the sensitivity of *r*_*max*_ estimation.

All *M. thurstoni* aged from Indonesian small-scale fisheries were less than six years old (*n* = 59), with the majority two years or younger (*n* = 49), primarily caught between September and January. Gillnets are generally selective for a narrower size range where the smallest individuals can swim through the net and the largest avoid become meshed and therefore captured (Harry et al. 2022). The larger offspring size of many rays and sharks often means they are vulnerable to capture, which would be the case for large devil ray offspring (Simpfendorfer 1999; Harry et al. 2022). Nevertheless, bycatch rates in gillnets are high for mobulids (Shahid et al. 2018; Fernando and Stewart 2021) and other elasmobranchs and marine megafauna more broadly (Lewison et al. 2004). Understanding gear selectivity is important for fisheries management to target specific species or size classes, adopt better fishing practices, and to implement effective bycatch mitigation (Thorpe and Frierson 2009; Harry et al. 2011; Braccini et al. 2022; Lemke and Simpfendorfer 2023). Selection for young *M. thurstoni* may also be due to temporal size segregation, which has been found for *M. thurstoni* in the Gulf of California (Notarbartolo di Sciara 1988). Limiting catch to sub-adults in a fishery whilst allowing adults to breed can be an effective management strategy (Prince 2002), yet protecting these age classes is also needed for the future reproductive output of the stock (Hixon et al. 2013; Kindsvater et al. 2016). Sub-adults likely have the largest influence on population growth and therefore our findings indicating that full recruitment to the fishery mainly occurs in sub-adults for both species before they have reached maturity is highly concerning for the population (Grant et al. 2020; Smart et al. 2020).

Growth coefficient estimates in this study are in line with larger-bodied chondrichthyans typically having low somatic growth rates, later maturity, and higher extinction risk (Cortés 2002). Yet, the study also provides initial evidence for geographic variation in devil ray life history. Our growth estimate for *M. mobular* was lower than published growth estimates for this species sampled off Mexico (Cuevas-Zimbrón et al. 2013; Pardo et al. 2016a). The Mexico size-at-age dataset had a similar size range to ours, with both lacking the largest size classes based on known maximum size for this species. Future sampling targeting these missing age classes (older and larger individuals) would be beneficial. *M. mobular* are reported to exhibit variation in size across their range (Marshall et al. 2022), which could result in growth differences. Estimates from both studies are still indicative of relatively slow growth for the species, which alongside the large body size of *M. mobular*, is associated with greater intrinsic sensitivity and higher extinction risk (Jennings et al. 1998; Reynolds et al. 2005). We found evidence for a later age-at-maturity (8 years) in Indian Ocean *M. mobular*, taking 2–3 years longer to mature compared to the same species off Mexico (5–6 years) (Cuevas-Zimbrón et al. 2013; Pardo et al. 2016a). This equates to a 15–20% reduction in lifetime reproductive output based on a maximum age of 20–26 years. Further, a delay in pregnancy from the onset of maturity has also been reported for this species, likely due to the large offspring size and long gestation period, where high maternal investment is needed (Rambahiniarison et al. 2018). Our estimates match closely with age-at-maturity estimates (7.4–9.1 years) reported from a study in the Philippines (Rambahiniarison et al. 2018) that used size-at-maturity estimates and the von Bertalanffy growth model from Cuevas-Zimbrón et al. (2013), with alternative model parameters (Cuevas-Zimbrón et al. 2013; Pardo et al. 2016a). We found a higher, yet still relatively low, growth coefficient estimate for *M. thurstoni* which is the first published estimate. We also present the first age-at-maturity estimate of seven years for *M. thurstoni*. Overall, this resulted in *r*_*max*_ estimates for both species that were comparable with previous estimates for *M. mobular* (median of 0.077 year^−1^, 95th percentiles 0.042, 0.108) (Pardo et al. 2016a), and manta rays (single estimate for *M. alfredi* and *M. birostris*) (median of 0.116 year^−1^, 95th percentiles 0.089, 0.139) (Dulvy et al. 2014), suggesting that Indian Ocean devil rays are at high risk of local depletion from overfishing.

Understanding species’ life history, alongside ecological and fisheries dynamics, can be key in informing the most effective actions to manage threats but there is uncertainty in the best sustainable management options for preventing species extinction (Denney et al. 2002; Sadovy 2005; Kindsvater et al. 2016). The conservative life history of devil rays makes it unlikely that they can withstand the fishing mortality rate found in this study (Stevens et al. 2000; Dulvy et al. 2008, 2014). Although there is substantial variation in the maximum size of devil rays between species, ranging from 110cm for *M. munkiana* to 700cm DW for *M. birostris* (Last et al. 2016), their low reproductive output limits their population growth rates. This is likely why consistently low *r*_*max*_ estimates have been found for devil rays with different maximum body sizes including for *M. mobular* and *M. thurstoni* in this study. Estimates of *r*_*max*_ are sensitive to the duration of the reproductive cycle (Dulvy et al. 2014), which is something that has only been reported in a handful of studies (Marshall and Bennett 2010; Rambahiniarison et al. 2018; Broadhurst et al. 2019). We aimed to account for uncertainty in all life history parameters used to estimate *r*_*max*_ through a Monte Carlo approach but variation in annual reproductive output and other parameters may lead to substantial variations in population growth rate that needs to be accounted for (Dulvy et al. 2014; Grant et al. 2020). Therefore, species- and region-specific life history estimates are key in informing accurate and localised demographic and sustainability assessments for devil rays (Dulvy et al. 2014).

Whilst devil rays are listed on CITES Appendix II and CMS Appendices I and II, national protections within the Indian Ocean are limited and the small-scale fisheries they are caught in typically have poor fisheries monitoring, regulation, and enforcement. This includes countries reporting some of the largest catch, such as Indonesia where we sampled in this study (Dulvy et al. 2014; Croll et al. 2016). Blanket bans on devil ray species as a sole management approach in small-scale fisheries would likely prove insufficient given the socio-economic dependence on these fisheries and could potentially lead to illegal trade. Thus, effective management needs to be tailored to the local context (Booth et al. 2019; Temple et al. 2024), with improved monitoring and enforcement mechanisms. Devil ray catches are often high value per individual and can contribute to a high proportion of the economic value of small-scale fisheries providing a financial incentive to exploit them (Temple et al. 2024). Small-scale fisheries are typically multi-gear and multi-species, making a management approach targeted towards a single species challenging (Herrón et al. 2019). Most devil ray catches occurred in gillnets and therefore management should prioritise interventions in these fisheries, such as complete bans in critical areas. However, this requires appropriate regulations, including international agreements, and capacity for enforcement, which is a challenge in the Indian Ocean region. It is crucial that well-designed protected areas are complemented by effective fisheries management to have the greatest impact for these economically valued and vulnerable species (Goetze et al. 2024). Management approaches could incorporate behavioural and attitudinal change of fishers to encourage the safe release of live-caught devil rays and reporting those entangled in gillnets (e.g. in Pakistan, fishers have been trained for safe handling and release of megafauna species including devil rays with a focus on target age/size classes), with many Regional Fisheries Management Organisations, including the Indian Ocean Tuna Commission, requiring live release, and recommending safe handling practices (IOTC 2019; Razzaque et al. 2020). Although gillnet discard mortality can be high (Dapp et al. 2016), there is some indication that mobulid rays may be capable of high post-release survival given the presence of a spiracle, depending on soak time and on board handling (Broadhurst and Cullis 2020). However, this can be challenging in small-scale fisheries where devil rays and other elasmobranchs caught incidentally are often utilised for subsistence and trade. Wider understanding of social and economic drivers of catch and fisher behaviour are therefore also needed for effective implementation of management actions (Barrowclift et al. 2017; Booth et al. 2023; Temple et al. 2024).

There is still insufficient life history data across the range of *M. mobular* and *M. thurstoni* to fully understand geographic differences as well as a lack of understanding of population structure. Gear selectivity may also mean that samples are not representative of the population, which could lead to biased growth estimates given the effects of fishing on the population (Walker et al. 1998; Thorson and Simpfendorfer 2009). The observed difference in growth for *M. mobular* between our study and the previous study of the same species off Mexico, could be partly due to a more limited number of individuals sampled in larger size classes in our dataset (DW > 2 m), whereby informative priors are still not “bending” the growth curve (a more bent curve results in faster doubling rates towards the asymptote and therefore a higher *k* estimate and concomitantly a lower *DW*_*∞*_ estimate). The lack of “bending” of the growth curve can also explain the unreasonably large estimates of *α*_*max*_ for *M. mobular* based on asymptotic size. Whilst we aimed to quantify any uncertainty and bias in age reads through human error, with commonly used techniques and the Bland–Altman approach, this error could have been carried through to our growth modelling (Harry et al. 2022). The age of larger and older individuals is likely underestimated (Harry 2018). We also assumed annual deposition of growth bands on vertebral centra but this could not be validated here and is not yet validated for any mobulid species, as with many elasmobranchs. Indeed, this may not be a valid assumption, with band pair deposition potentially being more variable (Harry 2018; Natanson et al. 2018; James and Natanson 2020). This can lead to additional uncertainty in age estimates as well as that of reader error that can be carried forward to subsequent analyses utilising length-at-age datasets (Harry et al. 2022).

Similar assumptions in catch curve analysis can also lead to potential biases in our total mortality estimate where they may not be met. These include no size selectivity in the catch, constant recruitment and natural mortality across age classes, a closed population, and sufficient sample size to represent the age structure of the population (Smith et al. 2012). Given that the largest individuals may not have been sampled in this study, our total mortality estimate may be higher leading to a greater fishing mortality estimate than if the full size range were sampled. Understanding the assumptions made and the limitations are important in appropriate use of life history estimates. Continued exploration or novel aging techniques are still needed given it is not possible to age all elasmobranch species due to vertebral morphology and lack of growth band pair formation (Burke et al. 2020). For the three devil ray species we aged, caudal vertebrae were the most calcified with clear banding of the vertebral column but this may need to be confirmed in further species. Difficulty in assessing female maturity and reproductive cycle as in this study is a common issue given low sample sizes across the year with the seasonality of many fisheries. A potential method that has been tested is the use of ultrasound (Froman et al. 2023), which would be useful for live and larger individuals as well as being a less destructive sampling method. It could also be a potential way to avoid the dissection of landed rays, which fishers and traders do not always agree to, making it difficult to collect female maturity and reproductive cycle data.

Given data deficiencies for devil rays and many other elasmobranchs, and the difficulty in addressing these gaps, data-poor methods need to be utilised with available information to ensure a precautionary approach to sustainable fisheries management can be taken until improved data becomes available. Low sample size is a common issue in elasmobranch age and growth studies. Uncertainty in maximum age estimates was high given we lacked larger individuals in our samples and was therefore estimated from the growth curves, which were slow to asymptote. Nonetheless, significantly reducing the range in *α*_*max*_ only marginally reduced *r*_*max*_ estimates. For example, reducing the *α*_*max*_ range from 17.5–64 to 17.5–26 for *M. thurstoni* only changed median *r*_*max*_ values from 0.107 yr^−1^ to 0.091 yr^−1^. Bayesian growth modelling can provide a useful alternative to fixing model parameters, which has been shown to bias growth estimates (Pardo et al. 2013), particularly when the smallest and largest age classes are lacking (Pardo et al. 2016a; Mukherji et al. 2021; Smart and Grammer 2021). We found both species had relatively slow growth, late age-at-maturity, low *r*_*max*_, and therefore high intrinsic sensitivity to fisheries exploitation (Cortés 2000, 2002; Reynolds et al. 2005). However, there is inter- and intra-specific variation in devil ray growth rates that warrants species- and population- estimates to inform more accurate species/population/stock assessment models. We demonstrate a suitable data-poor approach to generate age, growth, and *r*_*max*_ estimates for Endangered *M. mobular* and *M. thurstoni* to inform these assessments. Our findings reinforce previous works showing that devil rays can only withstand relatively low catch rates, which are almost certainly being outstripped by current targeted and incidental catch rates in small-scale and industrial fisheries across the Indian Ocean. Implementation of evidence-based fisheries management is critically needed for these species in Indian Ocean small-scale fisheries given their conservative life history and extinction risk status as well as their high ecological and socio-economic value.

## Supplementary Information

Below is the link to the electronic supplementary material.

Supplementary file1 (DOCX 1596 kb)

## Declarations

### Data and code availability

The data and code required to reproduce the analyses in this study are available at Github (https://github.com/EBarrowclift/LH-analyses.git).
