# Supplementary material for: Age, growth, and intrinsic sensitivity of Endangered Spinetail Devil Ray (Mobula mobular) and Bentfin Devil Ray (M. thurstoni) in the Indian Ocean
Source: Mar Biol. 2024 Dec 30;172(2):24. doi: 10.1007/s00227-024-04564-6 (PMC11885364; doi:10.1007/s00227-024-04564-6)
Supplement: Supplementary file 1 — Supplementary file1 (DOCX 1596 kb) [file 227_2024_4564_MOESM1_ESM.docx]

**Supplementary materials for the manuscript:**

**Age, growth, and intrinsic sensitivity of Endangered Spinetail Devil Ray (*Mobula mobular*) and Bentfin Devil Ray (*M. thurstoni*) in the Indian Ocean.**

**Ellen Barrowclift^1*^, Andrew J. Temple^2^, Sebastián A. Pardo^3^, Alexander M. A. Khan^4^, Shoaib Abdul Razzaque^5^, Nina Wambiji^6^, Mochamad Rudyansyah Ismail^4^, Lantun Paradhita Dewanti^4^, Per Berggren^1*^**

^1^School of Natural and Environmental Sciences, Newcastle University, Newcastle-upon-Tyne, United Kingdom.

^2^King Abdullah University of Science and Technology, Thuwal, Saudi Arabia.

^3^Pacific Biological Station, Fisheries and Oceans Canada, Nanaimo, BC, Canada.

^4^Faculty of Fisheries and Marine Sciences, Universitas Padjadjaran, Bandung, Indonesia.

^5^WWF Pakistan, Karachi, Pakistan.

^6^Kenya Marine and Fisheries Research Institute, Mombasa, Kenya.

Photo credit: WWF Pakistan

**
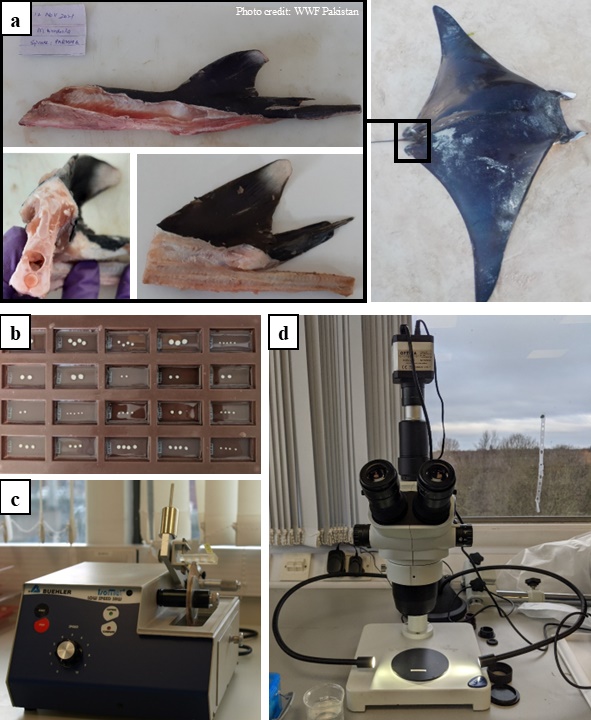
**

**Fig. S1.** Preparation of *Mobula* spp. vertebrae sections for age determination including: a) portion of the vertebral column where caudal vertebrae samples were taken; b) embedded vertebrae centra in an epoxy resin in silicon moulds; c) longitudinal sectioning of set vertebrae centra using a Buehler IsoMet Low-Speed Diamond Blade Saw fit with two 4 inch blades and a 3.5 inch 0.5mm plastic separator; and d) imaging of vertebrae sections for age determination using an Optika dissecting microscope with a fitted camera, illuminated from above using reflected light and from either side using a double-armed fibre optic light source.


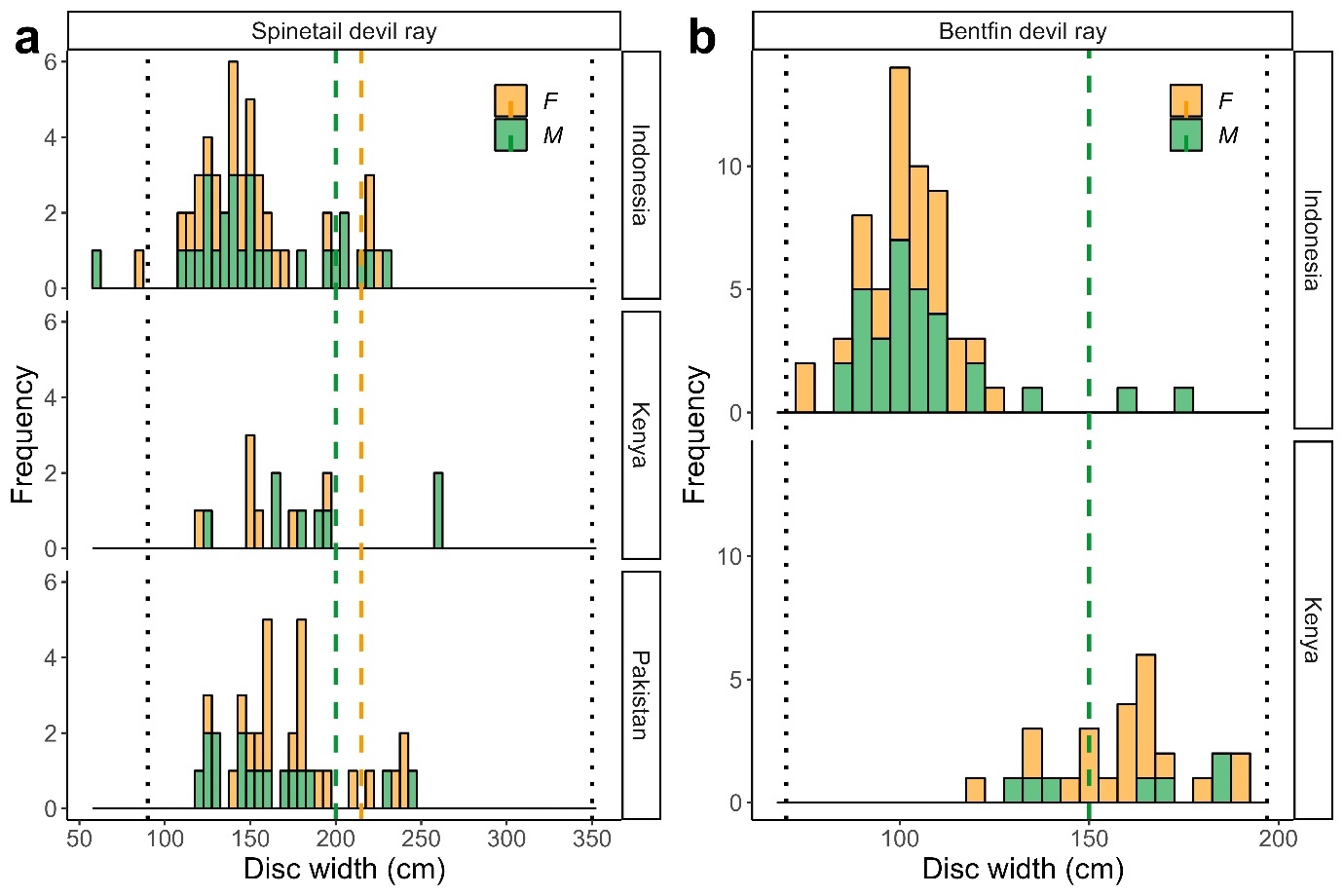


**Fig. S2.** Disc width (cm) frequency distribution for a) Spinetail devil ray (*M. mobular*) (*n=*103) and b) Bentfin devil ray (*M. thurstoni*) (*n=*89) sampled from Indonesia (*n=*112), Kenya (*n=*43), and Pakistan (*n=*37) with known female (orange dashed) and male (green dashed) minimum size at maturity; minimum offspring size and maximum size (black dotted lines) for each species indicated (IUCN, 2023).

**Table S1.** Age frequency for sampled Spinetail devil ray (*Mobula mobular*) (*n=*79) and Bentfin devil ray (*M. thurstoni*) (*n=*59) by location, sex, and month.

| **Species** | **Date** | **Indonesia** | **Pakistan** |
| --- | --- | --- | --- |
| *Mobula mobular* | January | 2 | - |
|  | March | - | 10 |
|  | May | 1 | - |
|  | June | - | 1 |
|  | July | 1 | - |
|  | September | 10 | 15 |
|  | October | 31 | 3 |
|  | November | 3 | 2 |
| *Mobula thurstoni* | January | 11 | - |
|  | September | 6 | - |
|  | October | 40 | - |
|  | November | 2 | - |

**Fig. S3.** Bland-Altman analyses of agreement, precision, and bias in age estimates (year ± 0.5) within and between readers for 1) Spinetail devil ray (*M. mobular*) (*n=*79) and 2) Bentfin devil ray (*M. thurstoni*) (*n=*59). Plots show the relationship between: vertebrae age band counts for a) reader 1 and c) reader 2 and between e) mean vertebrae age band counts from readers 1 and 2. b) Bland–Altman plots display bias and precision between vertebrae age band counts for b) reader 1 and d) reader 2 and between f) mean vertebrae age band counts from readers 1 and 2.


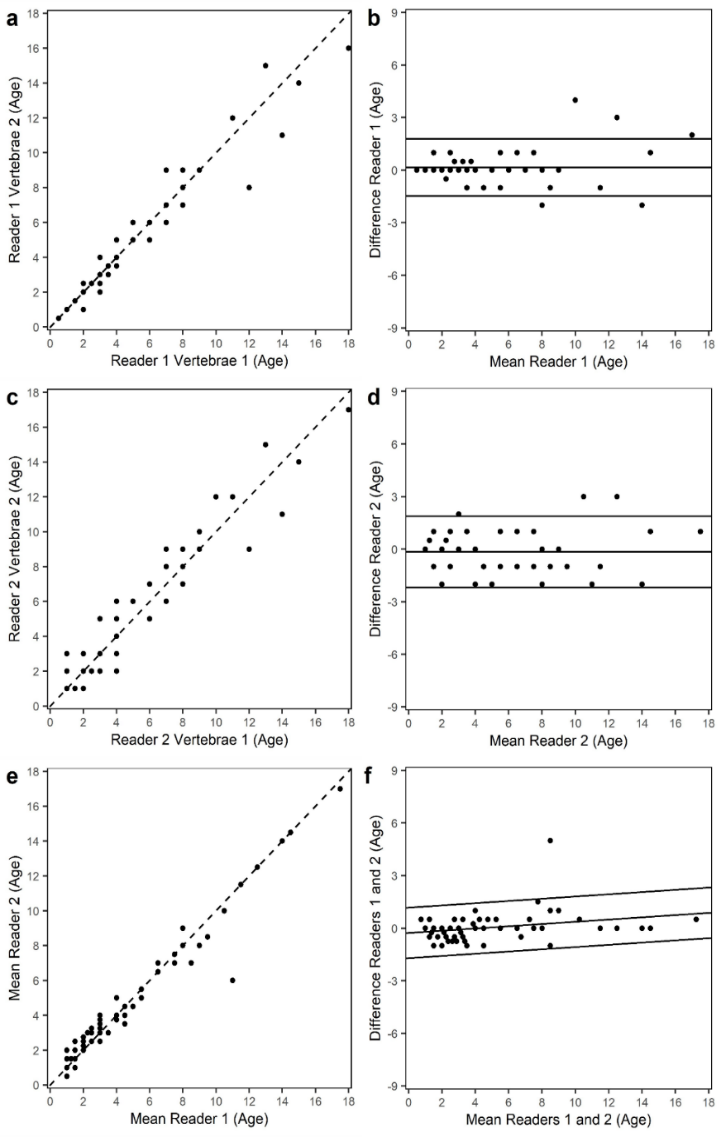

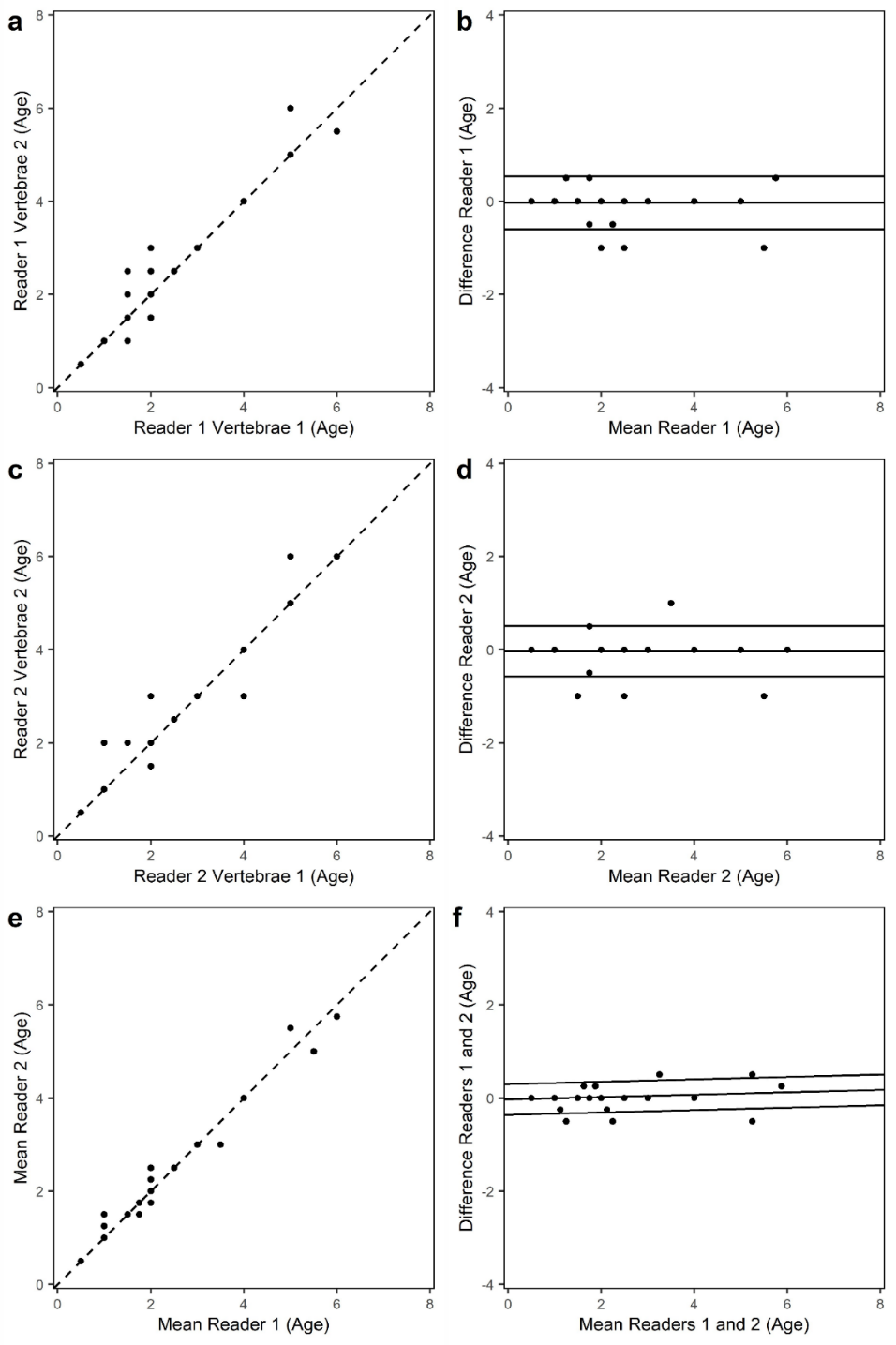


1. **Spinetail devil ray**
2. **Bentfin devil ray**
